# Parkinson Disease from Mendelian Forms to Genetic Susceptibility: New Molecular Insights into the Neurodegeneration Process

**DOI:** 10.1007/s10571-018-0587-4

**Published:** 2018-04-26

**Authors:** Amin Karimi-Moghadam, Saeid Charsouei, Benjamin Bell, Mohammad Reza Jabalameli

**Affiliations:** 1 Division of Genetics, Department of Biology, Faculty of Science, University of Isfahan, Isfahan, Iran; 2 Department of Neurology, Faculty of Medicine, Tabriz University of Medical Sciences, Tabriz, Iran; 3 Human Genetics & Genomic Medicine, Faculty of Medicine, Southampton General Hospital, University of Southampton, Southampton, UK

**Keywords:** Parkinson disease, Neurodegeneration, Autophagy, Mitochondrial dysfunction, Oxidative stress, GWAS meta-analysis

## Abstract

Parkinson disease (PD) is known as a common progressive neurodegenerative disease which is clinically diagnosed by the manifestation of numerous motor and nonmotor symptoms. PD is a genetically heterogeneous disorder with both familial and sporadic forms. To date, researches in the field of Parkinsonism have identified 23 genes or loci linked to rare monogenic familial forms of PD with Mendelian inheritance. Biochemical studies revealed that the products of these genes usually play key roles in the proper protein and mitochondrial quality control processes, as well as synaptic transmission and vesicular recycling pathways within neurons. Despite this, large number of patients affected with PD typically tends to show sporadic forms of disease with lack of a clear family history. Recent genome-wide association studies (GWAS) meta-analyses on the large sporadic PD case–control samples from European populations have identified over 12 genetic risk factors. However, the genetic etiology that underlies pathogenesis of PD is also discussed, since it remains unidentified in 40% of all PD-affected cases. Nowadays, with the emergence of new genetic techniques, international PD genomics consortiums and public online resources such as PDGene, there are many hopes that future large-scale genetics projects provide further insights into the genetic etiology of PD and improve diagnostic accuracy and therapeutic clinical trial designs.

## Introduction

Parkinson’s disease (PD) was first described by James Parkinson, an English doctor, in 1817 (Kempster et al. 2007). PD is known as a chronic, progressive neurodegenerative disease that affects 2% of the population over the age of 60 and 4% of the population over the age of 80 (late-onset PD). However, 10% of the disease can occur in younger adults, between 20 and 50 years of age (early-onset PD). Besides the age, several studies have found evidence of gender influence in the incidence of PD. It has been proven that PD is more prevalent in men than in women, with a ratio of 3:1, respectively; which may be attributable to the effect of estrogen on dopaminergic neurons and pathways in the brain (Schrag et al. 2000). PD is classically diagnosed by the manifestation of impaired motor function with an asymmetric onset that spreads with time to become bilateral. The majority motor impairments of PD arise owing to the dopaminergic neural loss in the substantia nigra pars compacta and the subsequent loss of dopamine input to forebrain (striatal) motor structures, leading to debilitating problems with tremor, muscular rigidity, and bradykinesia (slowness of movement) (Jankovic 2008). However, recent studies have recognized PD as a more complex disorder encompassing both motor (MS) and nonmotor symptoms (NMS). It has been proven that the occurrence of NMS is more prevalent among patients with PD and the frequency of them increases with the disease severity or during the course of the disease. Most patients with the long-term disease or severe pathology show 6–10 NMS. Also, there is increasing evidence that NMS such as sensory abnormalities (olfactory deficits), sleep disturbance (rapid eye movement), depression, autonomic dysfunction, and cognitive decline may precede the onset of motor signs of Parkinson’s disease (Jankovic 2008; O’sullivan et al. 2008). Therefore, NMS or premotor symptoms of the disease would be very informative for early diagnosis and identification of apparently normal older individuals with the full constellation of premotor signs and introducing neuroprotective strategies at an early stage in order to develop effective treatments for the disease (Berg et al. 2012; Stern et al. 2012).

Originally, PD has been identified as a genetically heterogeneous disorder which is classified into two genetic subtypes including monogenic familial forms with Mendelian inheritance and sporadic forms with no or less obvious familial aggregation. It has been proven that monogenic familial forms are caused by rare, highly penetrant pathogenic mutations; however, sporadic forms may result from contributions of environmental factors and genetic susceptibility (Davie 2008; De Lau and Breteler 2006; Lesage and Brice 2009; Taccioli et al. 2011). Now, considering the availability of high-throughput genetic analysis techniques and the access to large patient samples such as the International PD Genomics Consortium (IPDGC), the amount of information in the field of PD genetics in both areas is quickly growing. The aim of this review is to provide an overview of the recent genetic findings in both areas of familial and sporadic forms of PD disease.

## Familial PD

Researches in the field of Parkinsonism have reported that approximately 10% of all PD-affected cases typically tend to show a clear Mendelian inheritance pattern and familial aggregation associated with the high risk of PD recurrence (Hardy et al. 2009). Over the past decades, through the genetic studies in these families, at least 23 disease-segregating genes or loci causing various monogenic forms of PD have been identified so far (Table 1). The knowledge acquired from the protein products of these genes indicates that mitochondrial dysfunctions and impaired autophagy-based protein or organelle degradation pathways all play key roles in the neurodegeneration process within brain and pathogenesis of PD (Mullin and Schapira 2013; Ryan et al. 2015). Here, the genes implicated in Mendelian forms of PD are reviewed.

**Table 1:** Common familial Parkinson disease-associated genes and loci

Loci	Inheritance	Gene	Position	Protein	Disease onset	Mutations
PARK1	AD rarely sporadic	*SNCA*	4q21	Synuclein-alpha	Early onset rarely late onset	Missense; regulatory gene duplication or triplication
PARK2	AR sporadic	*PARKIN*	6q25–q27	E3 ubiquitin ligase	Early onset	Missense or nonsense; regulatory; splicing; small indels; deletions; insertions
PARK3	AD	Unknown	2p13	Unknown	Late onset	Unknown
PARK4	AD rarely sporadic	*SNCA*	4q21	Synuclein-alpha	Early onset rarely late onset	Missense; regulatory gene duplication or triplication
PARK5	AD	*UCHL1*	4p14	Ubiquitin C-terminal hydrolase L1	Late onset	Missense
PARK6	AR	*PINK1*	1p35–p36	PTEN-induced kinase	Early onset	Missense or nonsense; splicing; small indels; deletions; insertions
PARK7	AR	*DJ-1*	1p36	DJ-1	Early onset	Missense; regulatory; splicing; small indels; deletions; insertions
PARK8	AD sporadic	*LRRK2*	12q12	Leucine-rich repeat kinase 2	Late onset	Missense; splicing; small deletions
PARK9	AR	*ATP13A2*	1p36	Cation-transporting ATPase 13A2	Early onset	Missense; splicing; small indels; deletions; insertions
PARK10	Unclear	Unknown	1p32	Unknown	Unclear	Unknown
PARK11	AD	*GIGYF2*	2q36–q37	GRB10 interacting GYF protein 2	Late onset	Missense; small indels
PARK12	Unclear	Unknown	Xq21–q25	Unknown	Unclear	Unknown
PARK13	AD	*Omi*/*HTRA2*	2p13	Serine peptidase 2	Late onset	Missense; splicing
PARK14	AR	*PLA2G6*	22q12–q13	Phospholipase A2, group 6	Early onset	Missense; splicing; deletions; insertions
PARK15	AR	*FBXO7*	22q12–q13	F-box protein 7	Early onset	Missense; splicing
PARK17	AD	*VPS35*	16q11.2	Vacuolar protein sorting 35	Late onset	Missense; splicing
PARK18	AD	*EIF4G1*	3q27.1	Eukaryotic translation initiation factor 4 gamma, 1	Late onset	Missense; deletions; insertions
PARK19	AR	*DNAJC6*	1p31.3	DNAJ subfamily C member 6	Early onset	Missense or nonsense; splicing
PARK20	AR	*SYNJ1*	21q22.11	Synaptojanin-1	Early onset	Missense
PARK21	AD	*DNAJC13*	3q22.1	DNAJ subfamily C member 13	Early onset	Missense
PARK22	AD	*CHCHD2*	7p11.2	Coiled-coil-helix-coiled-coil-helix domain 2	Late onset	Missense
PARK23	AR	*VPS13C*	15q22.2	Vacuolar protein sorting 13C	Early onset	Missense; small deletion
–	AD for PD AR for GD	*GBA*	1q21	Glucocerebrosidase	Unclear	Missense; regulatory; splicing; small indels; deletions; insertions
–	AD	*SCA2*	12q24.1	Spinocerebellar ataxia type 2	Unclear	(CAG) three nucleotide repeat variations

## SNCA

*Synuclein-Alpha* (*SNCA*) was the first PD-associated gene to be identified and is inherited in an autosomal dominant manner (Polymeropoulos et al. 1996). Patients affected with *SNCA* mutations exhibit clinically late-onset and typical features of PD. However, several mutations have been identified to be associated with early-onset PD phenotypes and more severe features, including rapid progression of bradykinesia, rigidity and tremor, high prevalence of psychiatric symptoms, frequent dementia, prominent cognitive decline, autonomic dysfunctions, and moderate response to levodopa (l-3,4-dihydroxyphenylalanine; l-DOPA), which is a dopamine receptor agonist (Ibáñez et al. 2009; Lesage et al. 2013; Polymeropoulos et al. 1997). *SNCA* encodes a presynaptic protein (α-synuclein) and plays an important role in synaptic transmission (Liu et al. 2004). Several in vivo gene expression analyses have provided evidence for *SNCA* positive effects on synaptic vesicle recycling and mobilization in the proximity of axon terminal by its involvement in the regulation of phospholipase *D2* activity and induction of lipid droplet accumulation (Lotharius and Brundin 2002). Consistent with these analyses, some related experiments on animal models demonstrated that *SNCA* is associated with the synaptic plasticity by enhancing neurotransmitter release from the axon terminal (Nemani et al. 2010). In addition, several other studies have indicated the possible negative regulatory effect of *SNCA* on tyrosine hydroxylase activity, a rate-limiting enzyme in dopamine biosynthesis (Yu et al. 2004).

As illustrated in Table 1, to date, three classes of pathogenic mutations have been identified in *SNCA* gene: (1) missense point mutations in the coding region of *SNCA*, (2) dinucleotide repeat variation in the promoter region of *SNCA*, and (3) locus multiplications, including duplications and triplications, resulted from intra-allelic or inter-allelic unequal crossing over between Alu and LINE elements for segmental duplication, and both mechanisms for *SNCA* triplication. Quantitative gene expression analyses have proven that two last classes lead to pathogenic overexpression of the wild-type protein (Kojovic et al. 2012; Mutez et al. 2011).

**Fig. 1:**
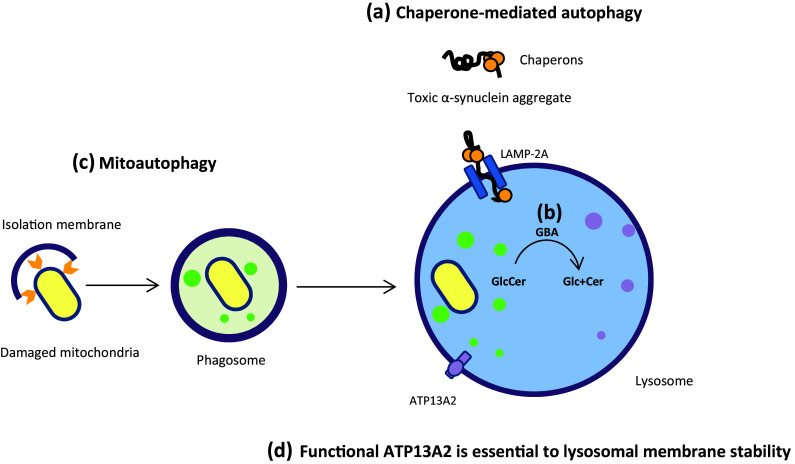
Lysosome-dependent degradation pathways; As indicated, **a** toxic α-synuclein aggregates are selectively degraded within the lysosome by means of LAMP-2A and chaperones; **b** GBA catalyzes the breakdown of sphingolipid glucosylceramide to ceramide and glucose within the lysosome; **c** damaged mitochondria is preferentially degraded by autophagosomal membrane engulfment and subsequent fusion with lysosome; **d** ATP13A2 is located inside the lysosomal membrane and its proper function is essential to the lysosomal membrane stability

*SNCA* mutations are suspected to have specific toxic effects in dopaminergic neurons. It seems that mutations in *SNCA* reduce the affinity of α-synuclein for lipids, thus increasing the tendency of the protein to form oligomers through a concentration-dependent mood, and consequently accelerate the formation of toxic α-synuclein fibrils (the major component of Lewy bodies) (Winner et al. 2011). It has been demonstrated that wild-type α-synuclein physically interacts with lysosome-associated membrane protein 2A (LAMP-2A), a transmembrane receptor for selective translocation of proteins into isolated lysosomes for the chaperone-mediated autophagy (CMA) pathway, providing support for the idea that CMA is involved in α-synuclein clearance (Fig. 1a). In fact, some pathogenic mutations in α-synuclein increase their affinity for LAMP-2A and act as uptake blockers, inhibiting both their own autophagy-dependent clearance and that of other CMA substrates. These studies provide another potential clue to the correlation of toxic gain of function mutations in α-synuclein with the lesions in PD (Cuervo et al. 2004; Wang and Mao 2014; Xilouri et al. 2016). Also, there is a hypothesis that a deficit in neurotransmitter release due to α-synuclein mutation could lead to cytoplasmic accumulation of dopamine, and increase oxidative stress and metabolic dysfunction in dopaminergic neurons (Lotharius and Brundin 2002), resulting from increased nonenzymatic and enzymatic oxidation of dopamine (Stefanis 2012). This finding has been corroborated by the Petrucelli et al. (2002) observations that mutant α-synuclein was selectively toxic to tyrosine hydroxylase positive neuroblastoma cells, but not in the neurons lacking tyrosine hydroxylase (Petrucelli et al. 2002).

### PARKIN

The second type of PD is caused by mutations in the *PARKIN* gene which leads to the autosomal recessive juvenile Parkinsonism (ARJP), the most prevalent known cause of early-onset (before age 45 years) PD (49% of familial early-onset PD and 15% of sporadic early-onset PD). Lücking et al. (2000) elucidated that there is a significant decline in the frequency of *PARKIN* mutations with increasing age at PD onset (Lücking et al. 2000). In particular, PD onset occurs before the age of 20, in 80% of patients with homozygous or compound heterozygous mutations in *PARKIN* gene (Klein et al. 2003; Mata et al. 2004; Periquet et al. 2003). It is now evident that mutations in *PARKIN* are associated with early development of motor symptoms, hyperreflexia, bradykinesia, dystonia, tremor, good response to low dose of l-DOPA at onset, and later l-DOPA-induced dyskinesia, as well as slow progression of psychiatric symptoms, with any clinical evidence of dementia (Ishikawa and Tsuji 1996; Ebba; Lohmann et al. 2003, 2009). Functionally, PARKIN is considered as a member of a multiprotein E3 ubiquitin ligase complex required for covalent attachment of activated ubiquitin molecules to target substrates (Shimura et al. 2000). This process is performed by a reaction cascade consisting of three groups of enzymes, including E1 ubiquitin-activating enzyme (UbA1), E2 ubiquitin-conjugating enzymes (UbCH7), and PARKIN E3 ubiquitin ligase (Pao et al. 2016; Trempe et al. 2013). The PARKIN-mediated ubiquitylation has various functional consequences, including the proteasomal degradation of misfolded or damaged proteins (Tanaka et al. 2004). It now appears that PARKIN also controls the mitochondrial quality through the selective lysosome-dependent degradation (autophagy or mitophagy) of dysfunctional mitochondria (Ryan et al. 2015).

As illustrated in Table 1, different types of mutations have been identified within *PARKIN* gene. Interestingly, it has proven that most of *PARKIN* mutation carriers have exon rearrangements in the heterozygous state (Stenson et al. 2017).

Mutations in *PARKIN* gene are associated with significant degeneration of dopaminergic neurons in the substantia nigra (Hristova et al. 2009). The presence of protein inclusions in Lewy bodies in PD patients led to the hypothesize that mutations in *PARKIN* cause a disruption in the E3 ubiquitin ligase activity of PARKIN, leading to insufficient clearance of damaged or mutated substrates and subsequent toxic cellular aggregation of unwanted proteins and neuronal cell death (Shimura et al. 2000). In addition, there is an idea that mutations in the *PARKIN* gene affect another important role of PARKIN in the turnover of mitochondria, reducing the ability of cells to remove damaged mitochondria by autophagy or mitophagy pathway (Pickrell and Youle 2015).

### PINK1

Homozygous or compound heterozygous mutations in *PTEN-induced kinase* (*PINK1*) gene are considered as the second leading cause of recessive early-onset PD (Valente et al. 2004). Clinically, patients with mutations in *PINK1* tend to present symptoms before the age of 40 and longer mean disease durations (Ibáñez et al. 2006). It has been described that the frequency of mutations varies between different populations from 1 to 15% (Nuytemans et al. 2010). Also, it has been proven that the clinical phenotype of PD appears to be broadly similar between patients with *PARKIN* and *PINK1* mutations, suggesting the idea that they might act together in pathways relevant to PD pathogenesis (Ibáñez et al. 2006). Interestingly, studies in Drosophila and mice also indicated a common PINK1/PARKIN pathway important for maintaining mitochondrial fidelity (Burman et al. 2012; Damiano et al. 2014; Moisoi et al. 2014; Park et al. 2006). Moreover, there are some indications that *PINK1* gene encodes a mitochondrial serine/threonine protein kinase and plays several important roles in mitochondrial pathways, including mitophagy, mitochondrial trafficking, and mitochondrial dynamics (Itoh et al. 2013; Narendra et al. 2010; Xinnan; Wang et al. 2011), which are largely consistent with the previous notion of PINK1/PARKIN common function in mitochondrial pathways.

Some mutations in *PINK1* may decrease the stability of the protein, whereas others significantly reduce the phosphorylation or kinase activity, supporting the hypothesis that mitochondrial dysfunction and oxidative stress may be associated with the PD (Deas et al. 2009; Gautier et al. 2008).

## PINK1, PARKIN, and Mitochondrial Hemostasis

Selective autophagic degradation of damaged mitochondria is necessary for mitochondrial homeostasis, an essential process for the cell survival (Franco-Iborra et al. 2016; McLelland et al. 2014). Cell biology studies revealed that PARKIN is selectively activated and recruited to depolarized mitochondria in order to drive damaged mitochondrial degradation (Vives-Bauza et al. 2010). PINK1 detects bioenergetically defective mitochondria, accumulates on it, and subsequently recruits PARKIN from the cytosol and instigates its E3 ubiquitin ligase activity by its kinase activity to trigger a cellular process for a selective degradation of mitochondria by autophagy (Kondapalli et al. 2012).

**Fig. 2:**
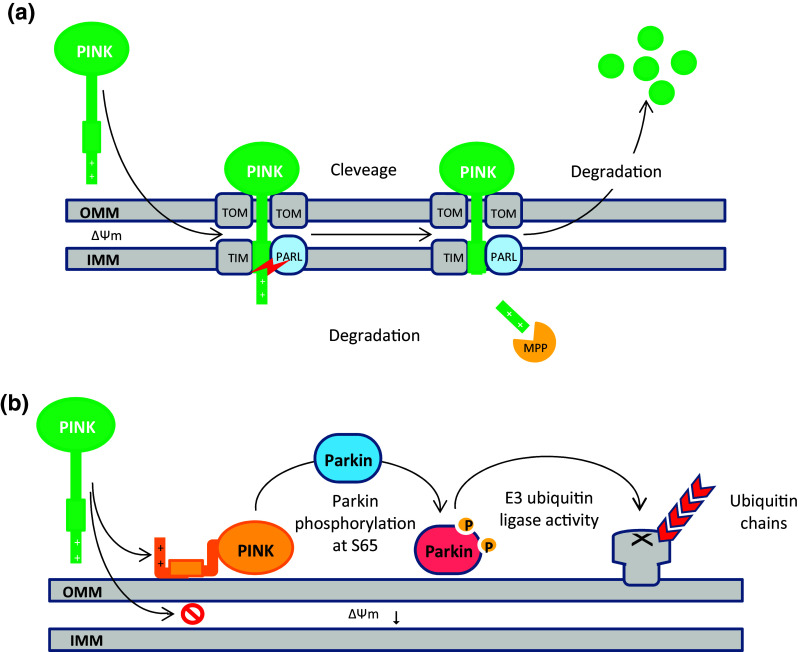
**a** Mitochondrial membrane potential (Δ*Ψ*) directs PINK1 towards OMM. PINK1 is continuously imported into mitochondria through the TOM/TIM complexes and subsequently targeting signal is cleaved and degraded by PARL and MPP, respectively. The truncated PINK1 is degraded by the ubiquitin proteasome system; **b** collapse of Δ*Ψ* blocks the TOM/TIM import pathway. PINK1 becomes stable on the OMM and recruits Parkin and activates its E3 ubiquitin ligase activity through the phosphorylation of Parkin on Ser65

PINK1 functions as a kind of molecular sensor, monitoring the internal state of individual mitochondria and flagging damaged mitochondria for removal (Matsuda et al. 2010). With respect to PINK1 roles in mitophagy, the damage-sensing mechanisms arise from the localization-dependent degradation of PINK1 in healthy mitochondria within a cell, which regulates PINK1 cytoplasmic concentration (Thomas et al. 2014). Under normal steady-state conditions, PINK1 is imported into the outer mitochondrial membrane (OMM) and thereby inner mitochondrial membrane (IMM), respectively, through the translocase of the outer membrane (TOM) and translocase of the inner membrane (TIM) complexes, cleaved by the IMM protease called Presenilin-associated rhomboid-like protein (PARL) and another mitochondrial processing peptidase (MPP), and subsequently degraded by the ubiquitin–proteasome system. This mechanism causes an undetectable concentration of PINK1 molecules on healthy mitochondria (Greene et al. 2012; Jin et al. 2010; Meissner et al. 2011). See Fig. 2a.

It has appeared that electrical component of the inner mitochondrial membrane potential (Δ*Ψ*) is crucial for the direction of PINK1 towards mitochondrial membrane and for its import into mitochondrial matrix compartment. The collapse of Δ*Ψ* blocks the TOM/TIM import pathway and in turn, prevents PARL/MPP rapid degradation mechanism causing PINK1 to accumulate uncleaved on the OMM, and binds to the outer mitochondrial membrane proteins such as TOM complex. When PINK1 becomes stable on the OMM, recruits PARKIN and activates its E3 ubiquitin ligase activity to enable OMM proteins polyubiquitination (Lazarou et al. 2012; Okatsu et al. 2013; Youle and Narendra 2011). Figure 2b shows that PINK1-mediated recruitment and activation of PARKIN occurs through Ser65 phosphorylation within the ubiquitin-like (Ubl) domain of PARKIN (Kazlauskaite et al. 2014). However, several recent biochemical investigations found that this process can be accelerated when PARKIN Ser65 phosphorylation combined with ubiquitin Ser65 phosphorylation (Kane et al. 2014). A model is presented for this positive feedback showing that phospho-ubiquitin generated by PINK1 (not unmodified ubiquitin) likely functions as an allosteric effector, binds to PARKIN allosteric site, and regulates its E3 ubiquitin ligase activity in a positive manner (Koyano et al. 2014). Once PARKIN is activated, it modifies various proteins on the OMM (36 substrates have been identified to date) and in the cytosol with K48- and K63-linked ubiquitin chains and thereby facilitates recruitment of specific autophagic receptor to ultimately degrade damaged mitochondria (Chan et al. 2011; Sarraf et al. 2013).

**Fig. 3:**
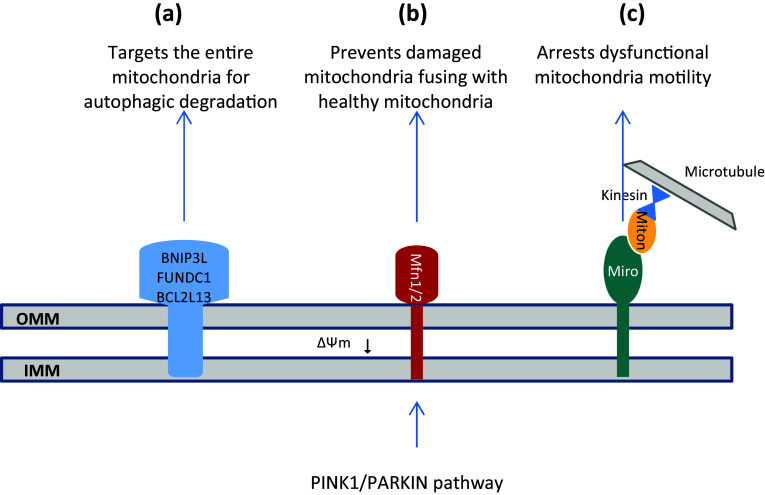
Schematic representation of three pathways that PINK1/PARKIN controls hemostasis of mitochondria; **a** PINK1/PARKIN pathway targets the entire mitochondria for autophagic degradation by attaching ubiquitin chains to some outer mitochondrial membrane (OMM) proteins; **b** PINK1/PARKIN pathway induces proteasomal degradation of Mfn1/2 and isolates dysfunctional mitochondria from the healthy mitochondria; **c** PINK1/PARKIN pathway releases Milton/Kinesin complex from mitochondrial surface through the proteasomal degradation of Miro1, and leading to arrest dysfunctional mitochondria motility

It has been reported that PINK1/PARKIN pathway facilitates mitophagy by altering mitochondrial trafficking (Xinnan Wang et al. 2011). Miro1 is a mitochondrial outer membrane protein that forms a complex with Milton and Kinesin to promote mitochondrial trafficking on microtubules (Boldogh and Pon 2007; Frederick and Shaw 2007). It has been demonstrated that PINK1 phosphorylates Miro1 on Ser156 to induce PARKIN and proteasomal degradation of it, releasing Milton/Kinesin complex from mitochondrial surface and leading to arrest dysfunctional mitochondria motility in neurons (Liu et al. 2012; Xinnan; Wang et al. 2011). This is considered as an initial quarantining step prior to mitophagy. See Fig. 3c.

Also, PINK1/PARKIN pathway appears to selectively affect the dynamics of dysfunctional mitochondria within the cell through the regulation of fusion/fission machinery as a mitochondrial quality control measure (Chen and Dorn 2013; Poole et al. 2008; Yu et al. 2015). In mammals, mitochondrial fusion was identified to be regulated by three membrane-bound GTPases, including mitofusins (Mfn) 1 and 2 for OMM fusion and optic atrophy 1 (OPA1) for IMM fusion (Chen et al. 2003; Song et al. 2007). PINK1 was reported to phosphorylate Mfn2 at Thr111 and Ser442 to induce PARKIN and subsequent proteasomal degradation of Mfn2 (Chen and Dorn 2013). It seems that PINK1/PARKIN pathway inhibits mitochondrial fusion through the degradation of Mfn1/2 and prevents damaged mitochondria fusing with healthy mitochondria. Such isolation of dysfunctional mitochondria from the healthy mitochondrial network is considered as an essential step prior to induction of mitophagy (Gegg et al. 2010; Poole et al. 2010). See Fig. 3b.

**Fig. 4:**
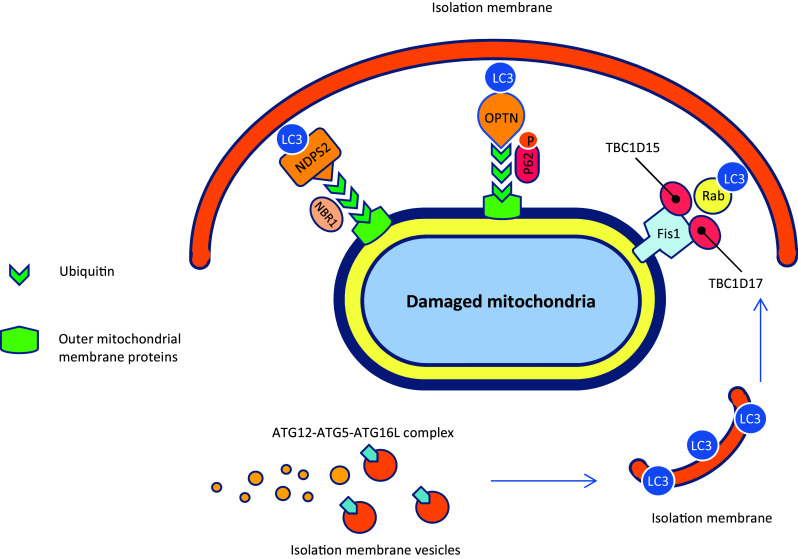
Schematic representation of the phagosome membrane formation around the damaged mitochondria. Refer to the text for explanations

Although PINK1/PARKIN pathway affects mitochondrial dynamics and trafficking by proteasomal degradation of specific mitochondrial outer membrane proteins (OMM proteins with K48-linked ubiquitin chains), it appears to target the entire mitochondria for autophagic degradation by selective recruitment of adaptor proteins to other mitochondrial outer membrane substrates (OMM proteins with K63-linked ubiquitin chains) (Narendra et al. 2012). There is a leading hypothesis that the ubiquitin chains attached by PARKIN to some OMM proteins or mitophagy receptors including BNIP3L (BCL2/adenovirus E1B 19 kDa protein-interacting protein 3-like), FUNDC1 (FUN14 domain-containing protein 1), and BCL2L13 (BCL2-like 13) serve as a positive signal for several different proteins such as p62/SQSTM1 (Sequestosome 1), NBR1 (Neighbor of BRCA1), NDP52 (Nuclear dot protein 52 kD), and OPTN (Optineurin) and recruit them to OMM (Gao et al. 2015; Geisler et al. 2010; Heo et al. 2015; Liu et al. 2012a, b; Otsu et al. 2015). These proteins function as adaptor proteins and bind both to ubiquitin chains and LC3/GABARAP (Gamma-aminobutyric acid receptor-associated protein) family members, which in turn recruit different protein complexes to growing isolation membranes that expand alongside mitochondria. The mechanisms involved in phagophore expansion are probably mediated by phagosome membrane uptake through the interaction of LC3/GABARAP with the autophagosome membrane and autophagy protein complex, ATG12-ATG5-ATG16L (Kabeya et al. 2004; Yang and Klionsky 2010). On the other hand, recent studies have uncovered that three mitochondrial localized proteins including RabGAPs, TBC1D15 (TBC1 Domain Family Member 15), and TBC1D17 (TBC1 Domain Family Member 17) bind to the mitochondrial outer membrane protein Fission1 via interaction with LC3/GABARAP and leads to positive regulation of autophagosomal membrane engulfment of mitochondria. The autophagosome then fuses with a lysosome, leading to degradation of the dysfunctional mitochondria by the proteases and lipases that reside in lysosomes (Shen et al. 2014; Yamano et al. 2014). See Figs. 1c, 3b, and 4.

### DJ-1

Mutations in the *DJ-1* gene are known to be associated with rare cases of autosomal recessive PD (1% of early-onset PD) (Bonifati et al. 2003). Clinically, patients affected with *DJ-1* mutations were found to have an early asymmetric development of dyskinesia, hyperreflexia, rigidity, and tremor, with later psychiatric symptoms including, psychotic disturbance, cognitive decline (uncommon), anxiety, and also a good response to l-DOPA (similar to clinical and phenotypic features of patients with *PARKIN* and *PINK1* mutations) (Abou-Sleiman et al. 2003; Annesi et al. 2005; Bonifati et al. 2003; Ibáñez et al. 2006). *DJ-1* encodes a protein involved in transcriptional regulation and antioxidative stress reaction within the neuronal cells (Ottolini et al. 2013). Under normal condition, subcellular localization investigations have revealed that DJ-1 is predominantly located in the cytoplasm and to a lesser extent in the nucleus and mitochondria within the neuronal cells (Junn et al. 2009; Nagakubo et al. 1997; Zhang et al. 2005). However, Junn et al. (2009) recently observed that DJ-1 translocation into the nuclear compartment is enhanced in response to oxidative stress (Junn et al. 2009). It has proven that the activation and subsequently nuclear localization of DJ-1 protects cells against reactive oxygen species (ROS), which is followed by self-oxidation at cysteine 106 (C106), a highly susceptible residue to oxidative stress (oxidative stress sensor residue), and formation of cysteine–sulfonic acid (SOH, SO_2_H) upon exposure to oxidative stress (Canet-Avilés et al. 2004; Kim et al. 2012; Kinumi et al. 2004). In addition, several studies have reported that under excessive oxidative stress conditions, DJ-1 is oxidized as SO_3_H at cysteine 46 (C46), cysteine 53 (C53), and cysteine 106 (C106) residues, which is an inactive form of DJ-1 observed in brains of patients with PD and Alzheimer’s disease (Bandopadhyay et al. 2004; Choi et al. 2006; Kinumi et al. 2004; Zhou et al. 2006).

In response to oxidative stress, DJ-1 in its oxidized form, acts as a neuroprotective transcriptional coactivator and regulates the activity of several DNA-binding transcription factors (TFs) including nuclear factor erythroid-2-like 2 (NFE2L2), polypyrimidine tract-binding protein-associated splicing factor (PSF) and p53 (Clements et al. 2006; Fan et al. 2008a, b; Zhong et al. 2006). Several lines of evidence obtained from separate studies suggesting that the TFs whose activity is regulated by DJ-1 may trigger multiple cytoprotective pathways against oxidative stress and subsequent neuronal cell death (Martinat et al. 2004; Venderova and Park 2012).

Investigation of ROS metabolism in human umbilical vein endothelial cells (HUVECs) has shown that NFE2L2 serves as a master TF for cellular antioxidant functions and detoxification responses (Kinumi et al. 2004). Without oxidative stresses, NFE2L2 is localized in the cytoplasm and interacts with KEAP1, which is an inhibitor protein and promotes ubiquitin–proteasome degradation of NFE2L2. Upon oxidative stress, DJ-1 disrupts the NFE2L2-KEAP1 interaction to stabilize NFE2L2, leading to translocation of NFE2L2 into the nucleus (Clements et al. 2006). This process is essential for the expression of several detoxifying and antioxidant enzyme genes through the binding of NFE2L2 to the antioxidant response elements (AREs) in their promoters, and thereby increasing neural protection against DNA damage and apoptosis (Im et al. 2012; Kensler et al. 2007; Vargas and Johnson 2009).

Tyrosine hydroxylase (TH) is a rate-limiting enzyme for dopamine synthesis and its deficiency contributes to the typical clinical symptoms of PD. Several protein-interaction studies have suggested that DJ-1 and PSF bind and transcriptionally regulate the human TH promoter (Ishikawa et al. 2009, 2010). Western blot analysis of SUMO species using immunoprecipitated PSF has demonstrated that PSF is sumoylated in human dopaminergic neuroblastoma SH-SY5Y cell lines. Sumoylation of PSF leads to the recruitment of histone deacetylase (HDAC) 1 to TH promoter and increase deacetylation of the TH promoter-bound histones, which subsequently results in the loss of TH expression and dopamine production. It has proven that DJ-1 positively regulates human TH gene expression by blocking the sumoylation of PSF and subsequently preventing HDAC1 recruitment to the TH promoter (Xu et al. 2005; Zhong et al. 2006). In addition, DJ-1 has been shown to stimulate vesicular monoamine transporter 2 (VMAT2) activities by transcriptional upregulation of VMAT2 gene and by direct binding to VMAT2 protein. VMAT2 is an integral membrane protein that transports cytosolic dopamine, a highly reactive molecule, into synaptic vesicles to avoid the effect of autoxidized dopamine on neuronal cell degeneration. These findings support the theory that stimulating activity of DJ-1 toward VMAT2 contributes to the protective reaction against dopamine toxicity (Ishikawa et al. 2012).

The p53 functions as a tumor suppressor protein and plays major roles in suppression of cell growth in response to stress conditions by induction of either cell cycle arrest or apoptosis. Human topoisomerase I-binding protein (Topors) is defined as a rate-limiting factor in the regulation of p53 activity. Under stress conditions, Topors acts as a coactivator of p53 and induces cell cycle arrest or apoptosis through enhancing the transcription of p53 downstream genes including Bax and p21 (Hofseth et al. 2004; Lin et al. 2005). DJ-1 has been shown to inhibit the induction of apoptosis by p53 through inhibition of Topors activity. It has also been reported that DJ-1 directly binds to the DNA-binding region of p53 and represses p53 transcriptional activity on Bax and p21 promoters, leading to neural cell cycle progression (Fan et al. 2008a, b; Kato et al. 2013).

It is suggested that DJ-1 involves within the cytoprotective pathways against oxidative stress and mutations in it cause the progressive apoptotic death of neuron cells, which can eventually lead to early onset of PD symptoms.

### LRRK2

Mutation in *Leucine-rich repeat kinase2* (*LRRK2*) gene is known as one of the common genetic cause of PD (Healy et al. 2008); they are responsible for at least 4% of autosomal dominant forms of familial PD typically associated with late onset and are also found in 1% of sporadic PD worldwide (Di Fonzo et al. 2005; Gilks et al. 2005; Nichols et al. 2005). Patients affected with *LRRK2* mutations exhibit a broad spectrum of clinical and phenotypic features including bradykinesia, muscular rigidity, tremor, cognitive decline, moderate dementia, olfactory deficits, hallucinations, sleep disturbance, orthostatic hypotension, and appreciable response to l-DOPA (Alcalay et al. 2009; Wszolek et al. 1995). However, several studies have reported that Lewy bodies (the pathological hallmarks of PD) are absent in some PD patients affected with *LRRK2* mutations (Funayama et al. 2005). The *LRRK2* gene encodes a large multifunction with important kinase activities. Some PD-associated mutations to *LRRK2* result in increased kinase activity of the protein, which may suggest a toxic gain of function mechanism. Wang et al. (2012) found that *LRRK2* regulates mitochondrial dynamics by interacting with a number of key regulators of mitochondrial fission/fusion, on mitochondrial membranes (Xinglong Wang et al. 2012). Wild-type *LRRK2* gene expression studies in human neuronal cell lines concluded that endogenous LRRK2 directly interacts with dynamin-related protein 1 (DRP1), a mitochondrial fission protein, increasing DRP1 phosphorylation and mitochondrial fission (Saez-Atienzar et al. 2014; Xinglong; Wang et al. 2012). The LRRK2-DRP1 interaction was enhanced by overexpressing wild-type *LRRK2* and by *LRRK2* PD-associated mutations (Su and Qi 2013; Xinglong; Wang et al. 2012). Also, it has been recently shown that LRRK2 modulates mitochondrial fusion regulators Mfn1/2 and OPA1 activities by interacting with them at the mitochondrial membrane. Additionally, decreased levels of reactive OPA1 have been observed in sporadic PD patients carrying some *LRRK2* pathogenic mutations (Stafa et al. 2013). Increased kinase activity of LRRK2 results in aberrant increased mitochondrial fragmentation which was associated with mitochondrial dysfunction, increased ROS production from mitochondrial complexes, and subsequently enhanced susceptibility to oxidative stress. These observations suggest that altered mitochondrial fission/fusion which is caused by mutations in *LRRK2* gene is an important factor in the pathogenesis of PD.

### HTRA2/OMI

*High-temperature requirement A2* (*HTRA2*/*OMI*) is another attractive candidate gene for PD that encodes a serine protease localizing to the mitochondrial intermembrane space (IMS). A heterozygous G399S missense mutation in the coding sequence of the gene was first identified in four German patients with PD (Strauss et al. 2005). However, evidence for the pathogenesis of *HTRA2*/*OMI* in PD has been further supported by whole exome sequence analyses in patients with PD from the Taiwan, Pakistan, Mexico, and in affected infants, born of consanguineous parents of Druze and Ashkenazi origins (Lin et al. 2011; Mandel et al. 2016; Oláhová et al. 2017). Also, some phenotypic similarities with parkinsonian features, including motor abnormalities and the progressive neurodegeneration in some brain regions, especially in the striatum were observed in HTRA2/OMI loss-of-function mice, indicating that HTRA2/OMI can serve a neuroprotective function (Jones et al. 2003; Martins et al. 2004). Loss of HTRA2/OMI protease activity in *OMI*-knockout mouse embryonic fibroblast cells showed increased mitochondrial DNA mutation, decreased mitochondrial membrane potential, altered mitochondrial morphology, and reduced mitochondrial density (Kang et al. 2013; Rathke-Hartlieb et al. 2002). It has been proposed that HTRA2/OMI is involved in the quality control of the proteins targeted for mitochondrial IMS by proteolysis of misfolded and damaged proteins, which is induced upon proteotoxic stress (Walle et al. 2008). In addition, it has been demonstrated that in mammalian cells HTRA2/OMI is released from mitochondria to the cytosol in response to apoptotic stimuli and induces apoptosis through interaction and proteolytic elimination of inhibitor of apoptosis proteins including c-IAP1 and XIAP (Suzuki et al. 2001; Yang et al. 2003). However, under nonapoptotic conditions, the HTRA2/OMI is restricted to the mitochondrial IMS and is also implicated in mitochondrial protein quality control (Cilenti et al. 2014; Kieper et al. 2010). These findings provided a link between mutations in *HTRA2*/*OMI* gene and mitochondrial dysfunction which is associated with neurodegeneration seen in some patients with PD (Bogaerts et al. 2008).

### CHCHD2

More recently, evidence for the role of mitochondrial dysfunction in the pathogenesis of Parkinson’s disease was further confirmed, based on the identification of heterozygous mutation in the *coiled-coil-helix-coiled-coil-helix domain 2 (CHCHD2)* gene using whole genome analysis in a Japanese family with autosomal dominant Parkinson disease. Clinical features of the patients usually include PD typical symptoms such as tremor, bradykinesia, rigidity, postural instability, and a good response to l-DOPA treatment (Funayama et al. 2015). This gene encodes a protein that is active in two cellular compartments including mitochondria and nucleus and is involved in the regulating mitochondrial metabolism under conditions of oxygen stress (Aras et al. 2015). In normal conditions, CHCHD2 is predominantly present within the mitochondrial intermembrane space (MIS) and binds to the subunit 4 of cytochrome C oxidase (COX4), which is necessary for optimal COX activity. COX is the last enzyme present in the electron transfer chain and plays a key role in the process of respiration within the mitochondrial membrane. In fact, its interaction with CHCHD2 plays a key role in maintaining energy balance inside the neurons under hypoxic conditions, by increasing COX4 efficiency and producing appropriate energy in the form of ATP via oxidative phosphorylation (Aras et al. 2013). Consistent with these observations, knockdown of CHCHD2 expression in human fibroblasts led to mitochondrial dysfunctions through reduced COX4 activity, oxygen consumption, and mitochondrial membrane potential, and increased ROS and mitochondrial fragmentation. Also, CHCHD2 functions as a master transcription factor to cope with oxidative stress. DNA-binding assays indicated that CHCHD2 binds to the proximal promoter of *COX4* gene as an oxygen responsive element (ORE) to increase its transcription. In addition, these studies revealed that CHCHD2 participates in a positive feedback loop and increases its expression through binding to ORE in its own promoter. It has been proven that although, a small portion of CHCHD2 is present in the nucleus under normal conditions, during the course of continuous oxidative stress the translocation of CHCHD2 into the nucleus is further stimulated in order to promote itself and COX gene transcription as anti-hypoxic responses (Aras et al. 2015, 2013). Furthermore, it has been reported that CHCHD2 binds to the Bcl-xL and regulates its activity in order to inhibit induction of apoptosis by the accumulation of Bax on the mitochondrial membrane under oxidative stress conditions (Liu et al. 2015). It is proposed that mutations in *CHCHD2* gene impair neuroprotection responses against hypoxic stress conditions through disruption of mitochondrial metabolism, thereby increasing the ROS level and also induction of apoptosis by Bax.

### VPS13C

Recently, whole genome studies in the field of Parkinsonism revealed that mutations in *vacuolar protein sorting 13C (VPS13C)* are associated with the development of autosomal recessive early-onset forms of PD. Clinically, patients affected with *VPS13C* mutations show the rapid and severe progression of bradykinesia, tremor, cognitive decline, and autonomic dysfunctions as well as a good response to l-DOPA treatment at the early stage (Lesage et al. 2016; Nalls et al. 2014). It has been proven that *VPS13C* encodes a member of a family of vacuolar protein sorting 13 (VPS13) (Velayos-Baeza et al. 2004). Currently, the molecular pathway(s) underlying how mutations in *VPS13C* cause PD *remain unknown*. However, in vitro experiments on human cell models showed that VPS13C is located on the outer mitochondrial membrane. Also, knockdown of *VPS13C* in the animal cell models is markedly associated with lower mitochondrial membrane potential, increased ROS, mitochondrial fragmentation, abnormal mitochondrial morphology, and upregulation of the expression of *PARKIN* and *PINK1* genes in response to toxin-induced mitochondrial dysfunction. It is believed that VPS13C cooperates with PARKIN/PINK1 pathway and contributes to the selective delivery of damaged mitochondria cargo to the lysosome (Lesage et al. 2016; Schreglmann and Houlden 2016). In fact, it is proposed that mutations in *VPS13C* gene may lead to the increased amount of ROS and dysfunctional mitochondria and ultimately trigger neuronal cell death.

### UCHL1

*Ubiquitin C-terminal hydrolase L1* (*UCHL1*) encodes a highly neuron-specific member of a gene family whose products function in the ubiquitin recycling pathway by hydrolyzing polymeric ubiquitin chains into monomers. The presence of UCHL1 in Lewy bodies and its function in the proteasome pathway suggested that it could be a compelling PD candidate gene. A heterozygous I93M mutation in the *UCHL1* gene was found in affected members of a German family with autosomal dominant Parkinson disease. Clinical manifestations such as tremor, muscular rigidity, bradykinesia, and postural instability, as well as good response to l-DOPA treatment, were typical for PD (Healy et al. 2004; Leroy et al. 1998). In vitro analysis showed that the mutant allele of *UCHL1* had ~50% reduced hydrolytic activity compared with the wild-type enzyme (Kensler et al. 2007; Nishikawa et al. 2003). Additionally, reduced levels of monoubiquitin in neurons were detected among the mice with neuroaxonal dystrophies, in which the function of UCHL1 was lost (Saigoh et al. 1999). However, in neuronal cell culture and mice, the expression of UCHL1 demonstrated an increase in the level of ubiquitin within the neurons (Osaka et al. 2003). These findings led to conclude that UCHL1 may play a role in ubiquitin stability within neurons, which is critical for ubiquitin–proteasome system and neuronal survival (Meray and Lansbury 2007).

### GBA

Several studies reported Parkinsonism in patients with Gaucher’s disease (GD), a lysosomal storage disorder caused by mutations in *Glucocerebrosidase* (*GBA*) gene (Grabowski 2008). Moreover, in some families affected with GD, several relatives of the probands developed Parkinsonism, many of whom were oblige heterozygous carriers of the *GBA* mutant alleles. The patients had an atypical onset of PD, including cognitive defects and hallucination. However, the disorder was progressive, and later they developed asymmetric manifestation of tremor, muscular rigidity, bradykinesia, and postural instability. It has been suggested that some *GBA* mutations may be a risk factor for the development of Parkinsonism in these families (Goker-Alpan et al. 2004; Sidransky 2004). The link between *GBA* and PD was also supported by neuropathology studies, showing dopaminergic neuronal dysfunction with widespread pathologies of α-synuclein and Lewy body in patients with homozygous and heterozygous *GBA* mutation (Kono et al. 2007). In addition, detailed biochemical studies showed significant decrease in glucocerebrosidase enzyme (GCase) activity and increase in α-synuclein accumulation in PD brains, with *GBA* mutations. GCase catalyzes the breakdown of sphingolipid glucosylceramide to ceramide and glucose within lysosomes and reduced enzyme activity and mutant protein may lead to impaired lysosomal protein degradation and increased exosomal release of α-synuclein and formation of its related toxic aggregates (Lin and Farrer 2014; Mazzulli et al. 2011; Schapira and Jenner 2011; Xu et al. 2011). See Fig. 1b. However, in line with these findings, most recent studies reported that the homozygous or heterozygous *GBA* mutations lead to a 20- to 30-fold increase in the risk of PD and 5–10% of PD patients have mutations in *GBA* gene (Velayati et al. 2010).

### ATP13A2

Originally, *ATPase type 13A2* (*ATP13A2*) has been reported associated with Kufor–Rakeb syndrome (KRS), which is a severe early-onset PD, inherited in an autosomal recessive manner. Clinically, patients affected with KRS tend to show progressive brain atrophy, tremor, rigidity, bradykinesia, dystonia, dementia, cognitive impairment, depression, supranuclear gaze palsy, and a better response to l-DOPA (Al-Din et al. 1994; Crosiers et al. 2011; Williams et al. 2005). *ATP13A2* gene belongs to the 5P-type subfamily of ATPase and encodes a lysosomal transmembrane protein that is mainly expressed in the brain. To date, the biochemical findings of ATP13A2 represent a class of proteins with unassigned function and substrate specificity (Dehay et al. 2012; Murphy et al. 2013; Ramirez et al. 2006). However, several different studies on the cultured KRS-patient dermal fibroblasts and other types of ATP13A2-deficient cell lines such as human neuroblastoma SHSY5Y cells determined that loss of functional ATP13A2 leads to instability of the lysosomal membrane and subsequently impaired lysosomal proteolysis function, which is essential to the lysosomal-mediated proper protein and mitochondrial quantity and quality control pathways within neurons (Dehay et al. 2012; Gusdon et al. 2012; Tofaris 2012); see Fig. 1d. These defects are tightly associated with pathogenic accumulation of α-synuclein and mitochondrial dysfunction, resulting in decreased ATP production and increased intracellular levels of ROS that contribute to the neuronal cell death (Gitler et al. 2009; Grünewald et al. 2012; Kong et al. 2014). In addition, several other studies have identified abnormal accumulation of manganese (Mn^2+^) and zinc (Zn^2+^) in the brain and cerebrospinal fluid of PD patients affected with ATP13A2 mutations (Fukushima et al. 2011; Hozumi et al. 2011; Jiménez-Jiménez et al. 1992). Moreover, Tan et al. (2011) found that overexpression of ATP13A2 in cultured neuronal cells exposed to Mn^2+^ reduced intracellular Mn^2+^ concentrations and protected cells from subsequent apoptosis (Tan et al. 2011). It is believed that ATP13A2 protects cells from metal toxicity by providing homeostasis of Mn^2+^ and Zn^2+^ (the significant environmental risk factors for PD) within neurons (Guilarte 2010; Pals et al. 2003; Rentschler et al. 2012).

It is speculated that mutations in ATP13A2 may disrupt normal intracellular homeostasis of divalent cations and lead to lysosomal and mitochondrial defects within neurons and ultimately significant neurodegeneration that is the distinguishing pathological feature of PD.

### PLA2G6

*Phospholipase A2 group 6 (PLA2G6)* has been characterized as the causative gene for different neurodegenerative diseases, including infantile neuroaxonal dystrophy (INAD), neurodegeneration with brain iron accumulation (NBIA), and Karak syndrome. However, recent genetic analysis of affected families from India, Iran, and Pakistan has been reported that mutations in the *PLA2G6* gene are responsible for early-onset dystonia-Parkinsonism with autosomal recessive inheritance (Morgan et al. 2006; Paisan-Ruiz et al. 2009; Paisán-Ruiz et al. 2010; Sina et al. 2009). The main clinical features of the patients affected with *PLA2G6* mutations are tremor, muscular rigidity, bradykinesia, dystonia, brain atrophy, dementia, visual disturbance, good response to l-DOPA therapy at first, and later l-DOPA-induced dyskinesia (Paisan-Ruiz et al. 2009; Sina et al. 2009; Yoshino et al. 2010). It has been proven that *PLA2G6* gene encodes calcium-independent group 6 phospholipase A2 enzyme, which hydrolyzes the *sn-2* ester bond of the membrane glycerophospholipids to yield free fatty acids and lysophospholipids (Balsinde and Balboa 2005). This function has profound effects on the repair of oxidative damage to the cellular and subcellular membrane phospholipids, membrane fluidity, and maintenance of membrane permeability or iron homeostasis (Balsinde and Balboa 2005; Shinzawa et al. 2008). In addition, Beck et al. (2015, 2016) demonstrated that knocking out the *PLA2G6* gene in mice leads to defects in remodeling of mitochondrial inner membrane and presynaptic membrane and subsequently causes mitochondrial dysfunction, age-dependent degeneration of dopamine nerve terminals, synaptic dysfunction, and significant iron accumulation in the brains of *PLA2G6* knockout mice (Beck et al. 2016, 2015). These findings suggest that impairment of the dopaminergic nervous system and brain iron accumulation caused by mutations in the *PLA2G6* gene can be considered as a pathogenic mechanism in sporadic and familial PD (Kauther et al. 2011).

### VPS35

**Fig. 5:**
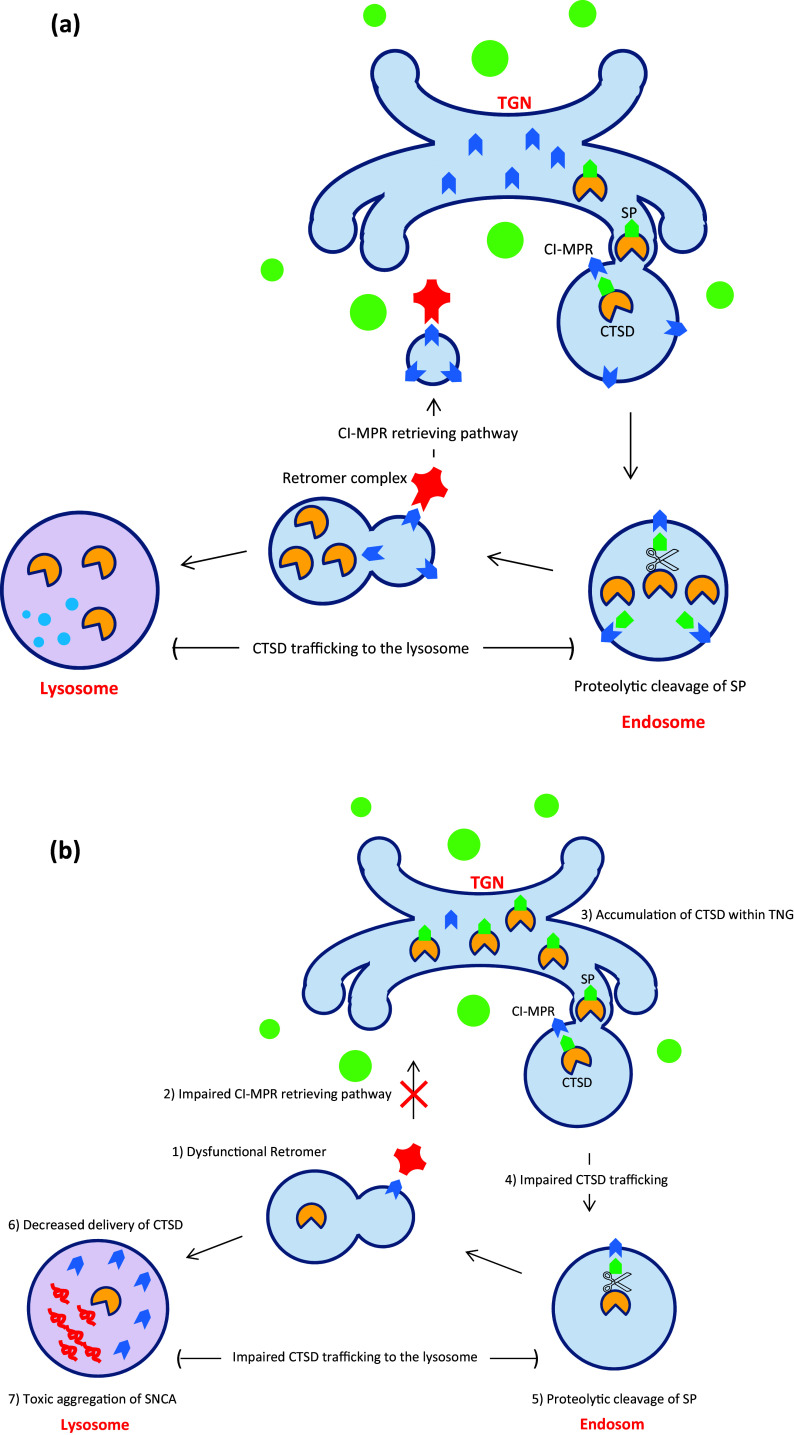
**a** VPS35 is a core component of the retromer cargo-recognition complex and plays a critical role in cargo retrieving pathway from the endosome to the trans-Golgi network (TGN); **b** mutations in VSP35 cause retromer complex dysfunction and lead to decreased delivery of CTSD to the lysosome and subsequently impaired lysosomal proteolysis function; Refer to the text for more explanations

In 2011, pathogenic mutations in the *vacuolar protein sorting 35* (*VPS35*) gene have been reported as novel causes of autosomal dominant PD, by application of whole exome sequencing to a large Swiss kindred representing late-onset tremor-predominant Parkinsonism (Vilariño-Güell et al. 2011). The main phenotypes associated with *VPS35* mutations in this kindred were tremor, dyskinesia, rigidity, dystonia, and good response to l-DOPA with rare cognitive or psychiatric symptoms (Kumar et al. 2012). Recent studies indicate that *VPS35* gene encodes a core component of the retromer cargo-recognition complex and plays a critical role in cargo retrieving pathway from the endosome to the trans-Golgi network (TGN) (Fuse et al. 2015; Tsika et al. 2014; Zavodszky et al. 2014). It has been proven that Cation-independent mannose 6-phosphate receptor (CI-MPR) is one of the best characterized cargo proteins of the retromer complex, which is involved in the trafficking of lysosomal proteases, such as the cathepsin D (CTSD), to lysosomes (Bugarcic et al. 2011; Choy et al. 2012; Seaman 2007). Under normal conditions, CTSD is specifically modified by attaching mannose 6 phosphates (M6P) residues to its signal peptide (M6P-CTSD) inside the TGN (Miura et al. 2014). Subsequently, M6P-CTSD is recognized by the CI-MPR and is trafficked from the TGN to the endosome. Inside the endosome, CTSD is activated by proteolytic cleavage of the signal peptide and then is released for further traffic to the lysosome. Ultimately, retromer retrieves free CI-MPRs from the endosome to the TGN, in which they can be involved in further cycles of CTSD trafficking to the lysosome (Laurent-Matha et al. 2006; Miura et al. 2014). It seems that dominant negative mutations in VSP35 cause retromer complex dysfunction and lead to decreased delivery of CTSD to the lysosome and subsequently impaired lysosomal proteolysis function which is essential to the lysosomal-mediated proper protein quality control pathways (Follett et al. 2014; Fuse et al. 2015; Hernandez et al. 2016). In addition, Miura et al. (2014) demonstrated that knocking down the *VPS35* gene in Drosophila leads to the toxic accumulation of the α-synuclein within the neurons which can further support the role of *VPS35* in the pathogenesis of PD (Miura et al. 2014). See Fig. 5.

### FBXO7

In 2008, *F-box protein 7* (*FBXO7*) was identified as a novel PD causative gene by a genome-wide linkage analysis in a large Iranian family, affected with autosomal dominant early-onset PD (Shojaee et al. 2008). Also, homozygote and compound heterozygote loss-of-function mutations in *FBXO7* have been reported in Italian and Dutch families. Affected members usually showed tremor, rigidity, bradykinesia, postural instability, hyperreflexia, saccadic eye movement with normal cognition, and appreciable response to l-DOPA (Di Fonzo et al. 2009a, b). To date, the precise mechanism by which FBXO7 contributes to neurodegeneration process remains poorly defined. However, it has been proven that FBXO7 functions as a molecular scaffold in the formation of protein complexes. FBXO7 has been reported to mediate the formation of SCF (Skp1, Cullin1, F-box protein) ubiquitin ligase complexes, and plays roles in the ubiquitin–proteasome degradation pathway (Nelson et al. 2013). In addition, recent invitro analyses have identified that FBXO7 physically interacts with PARKIN. In this regard, biochemical findings in Drosophila showed that overexpression of wild-type FBXO7 suppresses mitochondrial disruption and also neurodegeneration process in *PARKIN* mutants, confirming that they share a common role in mitochondrial biology (Burchell et al. 2013; Zhou et al. 2016). As a result, it is assumed that FBXO7 functions in a common pathway with PARKIN and PINK1 to induce selective autophagic clearance (mitophagy) in response to damaged mitochondria and pathogenic mutations in *FBXO7* may interfere with this pathway (Conedera et al. 2016; Randle and Laman 2017; Vingill et al. 2016).

### EIF4G1

Originally, mutations in *Eukaryotic translation initiation factor 4 gamma, 1* (*EIF4G1*) gene were identified in a large French family with autosomal dominant PD and confirmed in several families from the United States of America (USA), Canada, Ireland, Italy, and Tunisia. Clinically, affected individuals with *EIF4G1* mutations show late onset of asymmetric resting tremor, bradykinesia, muscle rigidity, with preserved cognition and good response to l-DOPA treatment (Chartier-Harlin et al. 2011). *EIF4G* gene family encodes a large scaffold protein that functions as a key initiation factor in mRNA translation and protein synthesis within eukaryotic cells by recruiting the multisubunit translation initiation factor complex at the 5′ cap of mRNAs (Ali et al. 2001). *EIF4GI* is a member of *EIF4G* gene family which selectively regulates the cap-dependent translation initiation of a subset of mRNAs encoding proteins function in mitochondrial activity, cellular bioenergetics, cellular growth, and proliferation in response to different cellular stresses (Ramírez-Valle et al. 2008; Silvera et al. 2009). Also, it has been reported that the high levels of EIF4GI are associated with malignancy in a significant number of human breast cancers suggesting that overexpression of EIF4GI may specifically increase cell proliferation and prevent autophagy in some human cancers (Schneider and Sonenberg 2007). Moreover, the loss of mitochondrial membrane potential and biogenesis has been observed in *EIF4GI*-silenced cells subjected to hydroperoxide treatment. It has been proposed that mutations in *EIF4G1* impair the mRNA translation initiation in PD. In fact, such mutations alter the translation of existing mRNAs essential to neuronal cell survival and their abilities to rapidly and dynamically respond to stress (Chartier-Harlin et al. 2011).

### GIGYF2

A genome-wide linkage analysis by use of 400 dinucleotide markers in a sample of sib pairs with late-onset autosomal dominant Parkinsonism found linkage to the 2q36–q37 chromosomal region (Pankratz et al. 2002). The marker with the highest linkage score (D2S206, LOD 5.14) was within the *Grb10-Interacting GYF Protein-2* (*GIGYF2*) gene region (Tan and Schapira 2010). Later sequence analysis of the *GIGYF2* gene region in 12 unrelated familial PD patients from Italy and France revealed seven different heterozygous mutations in the GIGYF2 gene, while these mutations were absent in controls (Lautier et al. 2008). However, there is some controversy surrounding the role of *GIGYF2* gene in the pathogenesis of PD, since several recent studies did not provide strong evidence for the association between *GIGYF2* gene mutations and PD (Bras et al. 2008; Di Fonzo et al. 2009b; Guo et al. 2009).

Studies in cultured cells, as well as yeast two-hybrid analysis, revealed that GIGYF2 may be recruited to activated-IGF-I/insulin receptors through binding to the N-terminus of Grb10 (Giovannone et al. 2003). Grb10 is recruited to tyrosine phosphorylated IGF-I/insulin receptors, in response to IGF-1/insulin stimulation (Dey et al. 1996; Hansen et al. 1996). It has been proven that Grb10 serves as an adaptor protein between NEDD4 and IGF-1 receptor and triggers ligand-induced ubiquitination and subsequent degradation of the IGF-I/insulin receptor (Langlais et al. 2004; Vecchione et al. 2003). Also, Overexpressing Grb10 gene in mice leads to postnatal growth retardation which further supports a role for the Grb10 protein in negatively regulating cell growth via the modulation of IGF-I/insulin receptor signaling (Dufresne and Smith 2005; Shiura et al. 2005). In contrast, expression of GIGYF2 in cultured cells showed a significant increase in IGF-1-stimulated receptor tyrosine phosphorylation (Higashi et al. 2010). In fact, it is postulated that GIGYF2 binding to Grb10 results in a significant increase in IGF-I/insulin receptor signaling pathway. In addition, a report showed that heterozygous GIGYF2+/− mice develop adult-onset neurodegeneration, indicating that *GIGYF2* gene dysfunction may have an important role in neurodegeneration process in the central nerve system (CNS) (Giovannone et al. 2003, 2009).

### ATXN2

During the last decade, researches in the field of Parkinsonism have described an association between CAG repeat expansions within the coding region of *Ataxin-2 (ATXN2)* gene and dominantly inherited familial forms of PD (Gwinn–Hardy et al. 2000; Payami et al. 2003). Molecular genetic analyses in affected families have reported that normal *ATXN2* alleles contain 14–31 CAG repeats, whereas pathologic alleles may carry expanded CAG repeats ranging in size from 35 to more than 200 (Lu et al. 2004). Clinical examinations suggest that cerebellar ataxia is usually the predominant symptom among patients. However, they often show some parkinsonian symptoms such as tremor, rigidity, bradykinesia, saccadic eye movement disorder, and good response to l-DOPA (Lu et al. 2004; Ragothaman et al. 2004). Although the biochemical function of ATXN2 is currently unknown, molecular studies in Drosophila suggest that ATXN2 may play roles in transport, stability, and translation regulation of a subset of mRNAs within neurons (Al-Ramahi et al. 2007; Halbach et al. 2015; Satterfield and Pallanck 2006). It seems that CAG repeat expansions within the coding sequences of *ATXN2*, resulting in the expansion of a polyglutamine (poly Q) tract in the ATXN2 may cause translational dysregulation of particular mRNAs and subsequently trigger the degeneration of dopaminergic neurons within the brain (Nkiliza et al. 2016; Satterfield and Pallanck 2006).

### DNAJC6

Autosomal recessive inheritance of mutations in the *DNAJC6* gene linked to juvenile-onset (< age 20) atypical Parkinsonism (PARK 19) has been reported. Disease progression in affected individuals was rapid, leading to a wheelchair-bound state within 10 years of onset. Response to l-DOPA was poor or absent and additional atypical manifestations such as mental retardation, seizures, dystonia, and pyramidal signs were observed (Edvardson et al. 2012; Koroglu et al. 2013). The *DNAJC6* gene codes for a brain-specific auxilin protein (Olgiati et al. 2016) which plays a role in the presynaptic endocytosis of clathrin-coated vesicles. The impairment of this pathway impacts on the formation of new vesicles at the presynaptic terminal (Kononenko and Haucke 2015). Variable phenotypes have been observed in PD patients expressing homozygous *DNAJC6* mutations with the onset of parkinsonian features occurring between the 3rd and 5th decade of life, disease progression being slower and with better responses to dopaminergic therapies. This separates patients markedly from PARK19 to be categorized as early-onset PD (< age 45) and suggests that some milder pathogenic mutations in the *DNAJC6* gene may allow for reduced auxilin expression (Olgiati et al. 2016).

### SYNJ1

Mutations in the *SYNJ1* gene have been reported to cause juvenile-onset atypical Parkinsonism (PARK20) through autosomal recessive inheritance. Typical features occurring at a young age include bradykinesia, tremor, dystonia, and apraxia of eyelid opening (ALO) as well as cognitive decline and generalized seizures in some patients (Quadri et al. 2013; Krebs et al. 2013; Olgiati et al. 2014). The *SYNJ1* gene encodes synaptojanin-1, a presynaptic phosphoinositide phosphatase protein which has a role in the regulation of synaptic vesicle endocytosis, important in the recycling of proteins. Animal study has shown that mutations in the Sac phosphatase domain of *SYNJ1* led to Parkinson’s-like neurological features and an increase in the levels of PD-associated proteins; auxilin, which has a similar role to synaptojanin-1in endocytosis and PARKIN. The impairment of the endocytic recycling pathway led to an accumulation of proteins at synaptic terminals and it was observed to selectively result in dystrophic dopaminergic axon terminals in the dorsal striatum. Phenotypic presentation in the animals studied provided strong evidence for a link between *SYNJ1* mutations and juvenile-onset PD, while elevated levels of auxilin and PARKIN suggesting an interaction with other PD-associated genes as a potential pathological mechanism (Cao et al. 2017).

### DNAJC13

The *DNAJC13* gene encodes an endosomal protein involved in clathrin coating of vesicles and as such is involved in intracellular transport. Mutations have been reported through a dominant inheritance leading to PD in patients, characterized by α-synuclein positive Lewy bodies, with age of onset being between 40 and 83 years. Disease progression is slow with duration noted at between 8 and 17 years and l-DOPA only effective in earlier stages (Vilarino-Guell et al. 2014; Appel-Cresswell et al. 2014; Gustavsson et al. 2015; Ross et al. 2016). It has been hypothesized that the accumulation of α-synuclein is a direct result of impaired intracellular transport due to toxic gain-of-function mutations in the *DNAJC13* gene. This has been demonstrated in vivo using Drosophila models which linked mutant *DNAJC13* to increased levels of insoluble α-synuclein in the fly head, degeneration of dopaminergic neurons, and age-dependent locomotor deterioration (Yoshida et al. 2018).

### PARK3, PARK 10, PARK 12

Several different genome-wide linkage analyses (GWLA) have been performed on the large groups of PD-affected families by genotyping of most popular genetic polymorphic markers including microsatellites and single-nucleotide polymorphisms (SNPs) (Funayama et al. 2015; Moghadam et al. 2017; Ott et al. 2015). Because PD is considered as a complex disease and causative loci may have different types of inheritance, the model of its inheritance is unknown (Keller et al. 2012). Therefore, linkage analysis based on model-free method would be more effective to map the loci responsible for the disease (Lander and Kruglyak 1995). In this approach, the PD-affected sibs inherited significantly more common alleles (identical by descent; IBD) at polymorphic loci linked to the disease than expected by chance (the expected probabilities of sharing 2, 1, and 0 IBD alleles for affected sib pairs at the disease locus will not be 0.25, 0.5, and 0.25, respectively) (Kruglyak et al. 1996; Nowak et al. 2012). As illustrated in Table 1, using model-free GWLA, three responsible loci for the PD have been mapped (*PARK3* on 2p13, *PARK10* on 1p32, and *PARK12* on Xq21-q25), but the causative genes have not yet been identified (DeStefano et al. 2002; Hicks et al. 2002; Pankratz et al. 2003).

## Sporadic PD

In the last decade, investigation of patients affected with PD has revealed that a large number of patients suffer from sporadic forms of PD, showing nonMendelian inheritance pattern of the disease and lack of a clear family history with no clear distinction in clinical symptoms or pathological signs from familial forms (Kalinderi et al. 2016; Verstraeten et al. 2015). Early candidate gene studies have revealed that only a small percentage of the sporadic PD cases carry mutations in a number of previously known Mendelian PD genes including *SNCA, PARKIN, LRRK2*, and *GBA1* (Table 1) (Maraganore et al. 2006; Satake et al. 2009; Zabetian et al. 2009). However, the etiology for a high proportion of sporadic PD cases remains largely unknown. It is assumed that the sporadic forms of PD are caused by the combined effects of common variations (polymorphisms with frequencies > 1%) in different genetic loci with minor to moderate effects on PD risk (average odds ratios (ORs) ~1.2) (Simon-Sanchez et al. 2009; Simón-Sánchez et al. 2011). In order to uncover the genetic architecture that impacts disease susceptibility in sporadic cases, more than 800 genome-wide association studies (GWAS) have been performed in the field of Parkinsonism during the last two decades, but most studies yielded inconsistent results. To alleviate this problem, GWAS meta-analysis has recently successfully been developed as a systematic approach to interpreting the genetic association findings of complex disease including neurodegenerative diseases (Consortium 2011; Evangelou et al. 2007). In addition, GWAS meta-analysis on 7,782,514 genetic variants in up to 13,708 PD cases and 95,282 controls from populations of European descent have been provided by a dedicated and freely available online database, PDGene (http://www.pdgene.org) (Lill et al. 2012). As illustrated in Table 2, twelve loci showed genome-wide significant association (ORs ≥ 1.1; *p* values < 5 × 10^−8^) with PD risk from case–control genotype data in 4 or more independent samples: *SNCA, TMEM175, STK39, TMEM229B, LRRK2, BCKDK, MIR4697, INPP5F, RIT2, GCH1, SIPA1L2, TMPRSS9* (Lill et al. 2012). However, despite this progress, the genetic etiology of PD, occurring in 40% of all cases remains unexplained by today (Consortium 2011).

**Table 2:** GWAS meta-analyses results of the PDGene database in the populations of European descent

Gene	Polymorphism	Location	Alleles	Case–control samples	Meta OR	Meta P-value
*SNCA* [− 19139 bp]	rs356182	chr4:90626111	G versus A	21	1.34	1.85e-82
*TMEM175*	rs34311866	chr4:951947	C versus T	21	1.26	6.00e-41
*STK39* [+ 24494 bp]	rs1955337	chr2:169129145	T versus G	21	1.21	1.67e-20
*TMEM229B*	rs1555399	chr14:67984370	T versus A	15	1.15	5.70e-16
*LRRK2*	rs76904798	chr12:40614434	T versus C	21	1.16	4.86e-14
*BCKDK*	rs14235	chr16:31121793	A versus G	21	1.10	3.63e-12
*MIR4697* [− 3032 bp]	rs329648	chr11:133765367	T versus C	21	1.11	8.05e-12
*INPP5F*	rs117896735	chr10:121536327	A versus G	13	1.77	1.21e-11
*RIT2*	rs12456492	chr18:40673380	G versus A	21	1.10	2.15e-11
*GCH1*	rs7155501	chr14:55347827	A versus G	15	1.12	1.25e-10
*SIPA1L2*	rs10797576	chr1:232664611	T versus C	21	1.13	1.76e-10
*TMPRSS9* [− 26450 bp]	rs62120679	chr19:2363319	T versus C	13	1.14	2.52e-09

## Discussion

It is increasingly evident that Parkinson’s disease (PD) is a complex and progressive neurodegenerative disorder clinically characterized by a broad spectrum of motor and nonmotor impairments. Over the past decades, both familial and sporadic forms of PD have been identified, with overlapping phenotypes. Family-based studies have successfully identified 23 loci or genes associated with PD. Subsequent functional characterization of the encoded proteins has revealed that lysosomal dysfunction, impaired mitophagy, deficiency of synaptic transmission, and vesicular recycling pathways can be considered as the key molecular mechanisms in spreading pathology of the disease that may be shared between familial and sporadic forms of PD. Accumulating evidence indicates that gene mutations lead to various abnormalities in one or several of these subcellular pathways and associate with neuronal loss in the substantia nigra pars compacta (SNc). Now, based on the pathological studies, degeneration of dopaminergic neurons in the SNc and subsequent reduction in the striatal concentration of dopamine are accepted as being responsible for spread of pathological features in both sporadic and familial PD (motor features of PD are mainly related to the dopamine deficit in the striatum, as dopamine plays a significant role in the control of motor function within brain) (Dickson et al. 2009). However, currently, there is no decisive description for why these disruptions affect dopaminergic neurons earlier and more profoundly than other neurons. One major common supposition for the selective vulnerability of SNc cells is the dopamine toxicity hypothesis. Dopamine metabolism is considered as a hot spot for the selective susceptibility of SNc cells to degeneration in PD (Segura-Aguilar et al. 2014). Dopamine metabolism produces highly reactive species and is vulnerable to different subcellular dysfunctions (Sulzer 2007). It is proposed that mitochondrial functional defects cause alterations in the mitochondrial respiratory chain as the main source of superoxide and hydrogen peroxide inside the neurons, and lead to the propagation of free radicals contributing to the oxidation of dopamine (Brieger et al. 2012). Also, deficiencies in the efficient elimination of damaged proteins or organelles (autophagy) due to impaired lysosome degradation pathway can lead to toxic protein aggregation and defective mitochondria accumulation inside the neuron which is associated with increased ROS formation as well as protein oxidation and enhanced vulnerability to oxidation of dopamine (Cook et al. 2012; Schapira et al. 2014). Moreover, it has been proven that reduced synaptic plasticity or impaired packaging of dopamine into the synaptic vesicles leads to an increased amount of cytosolic dopamine, which is readily susceptible to oxidation, and cause dopamine-mediated toxicity within the neurons (pH is lower inside the vesicles and dopamine cannot auto-oxidize) (Caudle et al. 2007; Zucca et al. 2014). Indeed, based on these observations, an emerging concept is that different gene mutations and subsequent mitochondrial dysfunctions, impaired lysosome degradation pathways, and reduced sequestration of dopamine into synaptic vesicles increase oxidative stress and interact with dopamine metabolism, which cause an exponential growth in the formation of highly reactive species of oxidized dopamine and precipitate lipid, protein, DNA, and other intracellular and membrane compounds oxidation as a critical step in the selective dopaminergic neuron death in the SNc over time (Jenner 2003; Segura-Aguilar et al. 2014).

In the past 30 years, this view that striatal dopamine loss secondary to degeneration of dopaminergic neurons might contribute to the pathogenesis of PD has guided the existing strategies for managing patients with PD and led to the development of dopamine replacement treatment using dopamine agonists (e.g., l-DOPA, ropinirole) and neuroprotective treatment (e.g., treatment with monoamine oxidase-B (MAO-B) inhibitors, glutamate antagonists, anti-apoptotic agents, growth factors) (Jenner 2004; Schapira 2009; Whone et al. 2003). Emerging evidence reveals that although dopaminergic treatment might provide some initial benefit in patients with PD, frequently lose antiparkinsonian efficacy, and develop levodopa-related motor complications and psychiatric manifestations, which means that many patients ultimately develop both motor and nonmotor problems (Parati et al. 1993; Schapira 2009). Recent knowledge offers cell replacement as a potential therapeutic opportunity for restoring striatal dopaminergic function in both familial and sporadic PD. It has been reported that Embryonic stem cells (ESCs) and induced pluripotent stem cells (iPSCs) may serve as promising sources of cells for transplantation in the striatum of PD patients (Björklund et al. 2002; Cai et al. 2009; Takahashi and Yamanaka 2006). Despite the fact that cell replacement studies have provided evidence for restoring motor functions in animal models of PD, to date, cell therapy efforts in PD patients have failed to show substantial clinical improvement and in some cases were hampered by the development of graft-induced dyskinesias (Cai et al. 2009; Politis et al. 2011). In addition, there is a considerable risk that they can overgrow and form teratoma after transplantation (Brederlau et al. 2006). More recently, gene therapy based on the adeno-associated viral vector (AAV)-mediated delivery of neuroprotective agents to the basal ganglia nuclei has provided a possible alternative approach to the conventional pharmacological treatments. It is now known that these gene therapy-based approaches failed in improving the motor symptoms in clinical trials and doubts about its benefits compared with existing drug treatment (Gasmi et al. 2007; Kaplitt et al. 2007; Lim et al. 2010). However, beyond these obstacles, currently, there is a general agreement that continued success in identifying the new genes implicated in the pathogenesis of PD is the best possible way to figure out what goes wrong at the molecular level and to use this knowledge to designing etiologic treatments for this complex disorder. In fact, it is clearly hoped that greater understanding of the genetic basis in inherited PD coupled with advancements in viral-mediated gene delivery may lead to potential gene replacement therapies and genetic defect corrections within the basal ganglia (etiologic gene therapy approach) (Büning et al. 2008; Singleton et al. 2013). In this context, several recent studies reported successful preclinical trials in multiple animal models based on the AAV-mediated delivery of *PARKIN* gene to the basal ganglia nuclei which reduced dopaminergic neurons degeneration and recovered motor functions (Manfredsson et al. 2007). Based on these findings, now, there is an incentive to broaden AAV-mediated gene replacement trials to other genetic defects associated with dopaminergic neuron degeneration, with this promising perspective that patients with different genetic defects may potentially benefit from gene replacement therapy in the future. Moreover, there is a common notion that understanding the potential mechanistic implications of these genes will broaden our options to design and produce efficient and specific drugs that appropriately intervene with the pathobiological process in both familial and sporadic PD (Singleton et al. 2013).

Additionally, with the advent of high-throughput genetic analysis techniques and the access to large patient samples, biomedical researches in the field of Parkinsonism have been radically changed. More recently, genome-wide association studies (GWASs) have been combined with meta-analysis and together have identified over 12 genetic risk factors. Ongoing researches demonstrated that these loci may be associated with increased risk for PD by affecting expression levels or splicing process of the biologically relevant transcripts (Consortium 2011; Simon-Sanchez et al. 2009). Currently, there is an assumption that identifying pathobiologically relevant transcripts within these risk loci and subsequently modulating their expression levels may provide novel potential therapeutic approaches for treating PD (Singleton et al. 2003). Also, aside from therapeutic interventions, it is worth mentioning that rapid progress in identifying the genes implicated either in the familial PD or in the sporadic PD as risk factors will be useful for diagnosing the disease in affected persons at an early stage and providing an opportunity to initiate appropriate therapeutic interventions at a presymptomatic stage in which a significant proportion of dopaminergic neurons are still alive and treatment is most likely to succeed. Moreover, considering the relationship between genetic variations within the risk loci and the level of gene expressions, it seems logical that genetic profiling of individuals affected with sporadic forms of the disease and also identifying the causative gene in patients affected with familial forms of the disease will be important in categorizing patients based on the pathogenicity mechanism and adopting appropriate treatment as well as the determining the drug dosage for treatments (Gibbs et al. 2010; Singleton et al. 2013).

Overall, given the genetically heterogeneous nature of the PD, elucidation of the genetic architecture of sporadic and familial PD improves diagnostic accuracy rates (sensitivity and specificity) and consequently enables presymptomatic diagnosis of the at-risk individuals as well as prenatal testing in the affected families. Moreover, it expands our knowledge of the disease genetic and neuropathologic mechanisms which can be of major importance for the development of disease-modifying therapeutic strategies. Ultimately, it enhances our ability to categorize various PD patients into genetic subtypes. This classification of patients based on the genetic etiology and underlying molecular mechanisms can pave the way for the efficient treatment of the patients through the effective intervention (slowing or halting) in the disease process.

## References

[CR1] Abou-Sleiman PM, Healy DG, Quinn N, Lees AJ, Wood NW (2003) The role of pathogenic DJ-1 mutations in Parkinson’s disease. Ann Neurol 54(3):283–286 12953260 10.1002/ana.10675

[CR2] Alcalay RN, Mejia-Santana H, Tang MX, Rosado L, Verbitsky M, Kisselev S et al (2009) Motor phenotype of LRRK2 G2019S carriers in early-onset Parkinson disease. Arch Neurol 66(12):1517–1522 20008657 10.1001/archneurol.2009.267 PMC2837584

[CR3] Al-Din A, Wriekat A, Mubaidin A, Dasouki M, Hiari M (1994) Pallido-pyramidal degeneration, supranuclear upgaze paresis and dementia: Kufor-Rakeb syndrome. Acta Neurol Scand 89(5):347–352 8085432 10.1111/j.1600-0404.1994.tb02645.x

[CR4] Ali IK, McKendrick L, Morley SJ, Jackson RJ (2001) Truncated initiation factor eIF4G lacking an eIF4E binding site can support capped mRNA translation. EMBO J 20(15):4233–4242 11483526 10.1093/emboj/20.15.4233 PMC149147

[CR5] Al-Ramahi I, Pérez AM, Lim J, Zhang M, Sorensen R, De Haro M et al (2007) dAtaxin-2 mediates expanded Ataxin-1-induced neurodegeneration in a Drosophila model of SCA1. PLoS Genet 3(12):e234 18166084 10.1371/journal.pgen.0030234 PMC2323314

[CR6] Annesi G, Savettieri G, Pugliese P, D’Amelio M, Tarantino P, Ragonese P et al (2005) DJ-1 mutations and parkinsonism-dementia-amyotrophic lateral sclerosis complex. Ann Neurol 58(5):803–807 16240358 10.1002/ana.20666

[CR7] Appel-Cresswell S, Rajput AH, Sossi V, Thompson C, Silva V, Mckenzie J et al (2014) Clinical, positron emission tomography, and pathological studies of DNAJC13 p.N855S parkinsonism. Mov Disord 29:1684–1687 25186792 10.1002/mds.26019

[CR8] Aras S, Pak O, Sommer N, Finley JrR, Hüttemann M, Weissmann N et al (2013) Oxygen-dependent expression of cytochrome c oxidase subunit 4–2 gene expression is mediated by transcription factors RBPJ, CXXC5 and CHCHD2. Nucleic Acids Res 41(4):2255–2266 23303788 10.1093/nar/gks1454 PMC3575822

[CR9] Aras S, Bai M, Lee I, Springett R, Hüttemann M, Grossman LI (2015) MNRR1 (formerly CHCHD2) is a bi-organellar regulator of mitochondrial metabolism. Mitochondrion 20:43–51 25315652 10.1016/j.mito.2014.10.003

[CR10] Balsinde J, Balboa MA (2005) Cellular regulation and proposed biological functions of group VIA calcium-independent phospholipase A 2 in activated cells. Cell Signal 17(9):1052–1062 15993747 10.1016/j.cellsig.2005.03.002

[CR11] Bandopadhyay R, Kingsbury AE, Cookson MR, Reid AR, Evans IM, Hope AD et al (2004) The expression of DJ-1 (PARK7) in normal human CNS and idiopathic Parkinson’s disease. Brain 127(2):420–430 14662519 10.1093/brain/awh054

[CR12] Beck G, Shinzawa K, Hayakawa H, Baba K, Yasuda T, Sumi-Akamaru H et al (2015) Deficiency of calcium-independent phospholipase A2 Beta induces brain iron accumulation through upregulation of divalent metal transporter 1. PLoS ONE 10(10):e0141629 26506412 10.1371/journal.pone.0141629 PMC4624760

[CR13] Beck G, Shinzawa K, Hayakawa H, Baba K, Sumi-Akamaru H, Tsujimoto Y et al (2016) Progressive axonal degeneration of nigrostriatal dopaminergic neurons in calcium-independent phospholipase A2β knockout mice. PLoS ONE 11(4):e0153789 27078024 10.1371/journal.pone.0153789 PMC4831782

[CR14] Berg D, Marek K, Ross GW, Poewe W (2012) Defining at-risk populations for Parkinson’s disease: Lessons from ongoing studies. Mov Disord 27(5):656–665 22508284 10.1002/mds.24985

[CR15] Björklund LM, Sánchez-Pernaute R, Chung S, Andersson T, Chen IYC, McNaught KSP et al (2002) Embryonic stem cells develop into functional dopaminergic neurons after transplantation in a Parkinson rat model. Proc Natl Acad Sci USA 99(4):2344–2349 11782534 10.1073/pnas.022438099 PMC122367

[CR16] Bogaerts V, Nuytemans K, Reumers J, Pals P, Engelborghs S, Pickut B et al (2008) Genetic variability in the mitochondrial serine protease HTRA2 contributes to risk for Parkinson disease. Hum Mutat 29(6):832–840 18401856 10.1002/humu.20713

[CR17] Boldogh IR, Pon LA (2007) Mitochondria on the move. Trends Cell Biol 17(10):502–510 17804238 10.1016/j.tcb.2007.07.008

[CR18] Bonifati V, Rizzu P, van Baren MJ, Schaap O, Breedveld GJ, Krieger E et al (2003) Mutations in the DJ-1 gene associated with autosomal recessive early-onset parkinsonism. Science 299(5604):256–259 12446870 10.1126/science.1077209

[CR19] Bras J, Simón-Sánchez J, Federoff M, Morgadinho A, Januario C, Ribeiro M et al (2008) Lack of replication of association between GIGYF2 variants and Parkinson disease. Hum Mol Genet 18(2):341–346 18923002 10.1093/hmg/ddn340 PMC2638775

[CR20] Brederlau A, Correia AS, Anisimov SV, Elmi M, Paul G, Roybon L et al (2006) Transplantation of human embryonic stem cell-derived cells to a rat model of Parkinson’s disease: effect of in vitro differentiation on graft survival and teratoma formation. Stem Cells 24(6):1433–1440 16556709 10.1634/stemcells.2005-0393

[CR21] Brieger K, Schiavone S, Miller FJ Jr, Krause KH (2012) Reactive oxygen species: from health to disease. Swiss Med Wkly 142:w13659 22903797 10.4414/smw.2012.13659

[CR22] Bugarcic A, Zhe Y, Kerr MC, Griffin J, Collins BM, Teasdale RD (2011) Vps26A and Vps26B subunits define distinct retromer complexes. Traffic 12(12):1759–1773 21920005 10.1111/j.1600-0854.2011.01284.x

[CR23] Büning H, Perabo L, Coutelle O, Quadt-Humme S, Hallek M (2008) Recent developments in adeno-associated virus vector technology. J Gene Med 10(7):717–733 18452237 10.1002/jgm.1205

[CR24] Burchell VS, Nelson DE, Sanchez-Martinez A, Delgado-Camprubi M, Ivatt RM, Pogson JH et al (2013) The Parkinson’s disease-linked proteins Fbxo7 and Parkin interact to mediate mitophagy. Nat Neurosci 16(9):1257–1265 23933751 10.1038/nn.3489 PMC3827746

[CR25] Burman JL, Yu S, Poole AC, Decal RB, Pallanck L (2012) Analysis of neural subtypes reveals selective mitochondrial dysfunction in dopaminergic neurons from parkin mutants. Proc Natl Acad Sci USA 109(26):10438–10443 22691499 10.1073/pnas.1120688109 PMC3387060

[CR26] Cai J, Yang M, Poremsky E, Kidd S, Schneider JS, Iacovitti L (2009) Dopaminergic neurons derived from human induced pluripotent stem cells survive and integrate into 6-OHDA-lesioned rats. Stem cells Dev 19(7):1017–1023 10.1089/scd.2009.0319 PMC3135248 19824823

[CR27] Canet-Avilés RM, Wilson MA, Miller DW, Ahmad R, McLendon C, Bandyopadhyay S et al (2004) The Parkinson’s disease protein DJ-1 is neuroprotective due to cysteine-sulfinic acid-driven mitochondrial localization. Proc Natl Acad Sci USA 101(24):9103–9108 15181200 10.1073/pnas.0402959101 PMC428480

[CR28] Cao M, Wu YM, Ashrafi G, Mccartney AJ, Wheeler H, Bushong EA et al (2017) Parkinson Sac domain mutation in synaptojanin 1 impairs clathrin uncoating at synapses and triggers dystrophic changes in dopaminergic axons. Neuron 93:882–896 28231468 10.1016/j.neuron.2017.01.019 PMC5340420

[CR29] Caudle WM, Richardson JR, Wang MZ, Taylor TN, Guillot TS, McCormack AL et al (2007) Reduced vesicular storage of dopamine causes progressive nigrostriatal neurodegeneration. J Neurosci 27(30):8138–8148 17652604 10.1523/JNEUROSCI.0319-07.2007 PMC6672727

[CR30] Chan NC, Salazar AM, Pham AH, Sweredoski MJ, Kolawa NJ, Graham RL et al (2011) Broad activation of the ubiquitin-proteasome system by Parkin is critical for mitophagy. Hum Mol Genet ddr048 10.1093/hmg/ddr048 PMC3071670 21296869

[CR31] Chartier-Harlin MC, Dachsel JC, Vilariño-Güell C, Lincoln SJ, Leprêtre F, Hulihan MM et al (2011) Translation initiator EIF4G1 mutations in familial Parkinson disease. Am J Hum Genet 89(3):398–406 21907011 10.1016/j.ajhg.2011.08.009 PMC3169825

[CR32] Chen Y, Dorn GW (2013) PINK1-phosphorylated mitofusin 2 is a Parkin receptor for culling damaged mitochondria. Science 340(6131):471–475 23620051 10.1126/science.1231031 PMC3774525

[CR33] Chen H, Detmer SA, Ewald AJ, Griffin EE, Fraser SE, Chan DC (2003) Mitofusins Mfn1 and Mfn2 coordinately regulate mitochondrial fusion and are essential for embryonic development. J Cell Biol 160(2):189–200 12527753 10.1083/jcb.200211046 PMC2172648

[CR34] Choi J, Sullards MC, Olzmann JA, Rees HD, Weintraub ST, Bostwick DE et al (2006) Oxidative damage of DJ-1 is linked to sporadic Parkinson and Alzheimer diseases. J Biol Chem 281(16):10816–10824 16517609 10.1074/jbc.M509079200 PMC1850953

[CR35] Choy RWY, Cheng Z, Schekman R (2012) Amyloid precursor protein (APP) traffics from the cell surface via endosomes for amyloid β (Aβ) production in the trans-Golgi network. Proc Natl Acad Sci USA 109(30):E2077-E2082 22711829 10.1073/pnas.1208635109 PMC3409748

[CR36] Cilenti L, Ambivero CT, Ward N, Alnemri ES, Germain D, Zervos AS (2014) Inactivation of Omi/HtrA2 protease leads to the deregulation of mitochondrial Mulan E3 ubiquitin ligase and increased mitophagy. BBA-Mol Cell Res 1843(7):1295–1307 10.1016/j.bbamcr.2014.03.027 24709290

[CR37] Clements CM, McNally RS, Conti BJ, Mak TW, Ting JPY (2006) DJ-1, a cancer-and Parkinson’s disease-associated protein, stabilizes the antioxidant transcriptional master regulator Nrf2. Proc Natl Acad Sci USA 103(41):15091–15096 17015834 10.1073/pnas.0607260103 PMC1586179

[CR38] Conedera S, Apaydin H, Li Y, Yoshino H, Ikeda A, Matsushima T et al (2016) FBXO7 mutations in Parkinson’s disease and multiple system atrophy. Neurobiol Aging 40:e191–e195 10.1016/j.neurobiolaging.2016.01.003 26882974

[CR39] Consortium IPDG. (2011) Imputation of sequence variants for identification of genetic risks for Parkinson’s disease: a meta-analysis of genome-wide association studies. Lancet 377(9766):641–649 21292315 10.1016/S0140-6736(10)62345-8 PMC3696507

[CR40] Cook C, Stetler C, Petrucelli L (2012) Disruption of protein quality control in Parkinson’s disease. Cold Spring Harb Perspect Med 2(5):a009423 22553500 10.1101/cshperspect.a009423 PMC3331692

[CR41] Crosiers D, Ceulemans B, Meeus B, Nuytemans K, Pals P, Van Broeckhoven C et al (2011) Juvenile dystonia-parkinsonism and dementia caused by a novel ATP13A2 frameshift mutation. Parkinsonism Relat Disord 17(2):135–138 21094623 10.1016/j.parkreldis.2010.10.011

[CR42] Cuervo AM, Stefanis L, Fredenburg R, Lansbury PT, Sulzer D (2004) Impaired degradation of mutant α-synuclein by chaperone-mediated autophagy. Science 305(5688):1292–1295 15333840 10.1126/science.1101738

[CR43] Damiano M, Gautier CA, Bulteau AL, Ferrando-Miguel R, Gouarne C, Paoli MG et al (2014) Tissue-and cell-specific mitochondrial defect in Parkin-deficient mice. PLoS ONE 9(6):e99898 24959870 10.1371/journal.pone.0099898 PMC4069072

[CR44] Davie CA (2008) A review of Parkinson’s disease. Br Med Bull 86(1):109–127 18398010 10.1093/bmb/ldn013

[CR45] De Lau LM, Breteler MM (2006) Epidemiology of Parkinson’s disease. Lancet Neurol 5(6):525–535 16713924 10.1016/S1474-4422(06)70471-9

[CR46] Deas E, Plun-Favreau H, Wood NW (2009) PINK1 function in health and disease. EMBO Mol Med 1(3):152–165 20049715 10.1002/emmm.200900024 PMC3378127

[CR47] Dehay B, Ramirez A, Martinez-Vicente M, Perier C, Canron MH, Doudnikoff E et al (2012) Loss of P-type ATPase ATP13A2/PARK9 function induces general lysosomal deficiency and leads to Parkinson disease neurodegeneration. Proc Natl Acad Sci USA 109(24):9611–9616 22647602 10.1073/pnas.1112368109 PMC3386132

[CR48] DeStefano AL, Lew MF, Golbe LI, Mark MH, Lazzarini AM, Guttman M et al (2002) PARK3 influences age at onset in Parkinson disease: a genome scan in the GenePD study. Am J Hum Genet 70(5):1089–1095 11920285 10.1086/339814 PMC447587

[CR49] Dey B, Frick K, Lopaczynski W, Nissley S, Furlanetto R (1996) Evidence for the direct interaction of the insulin-like growth factor I receptor with IRS-1, Shc, and Grb10. Mol Endocrinol 10(6):631–641 8776723 10.1210/mend.10.6.8776723

[CR50] Di Fonzo A, Rohé CF, Ferreira J, Chien HF, Vacca L, Stocchi F et al (2005) A frequent LRRK2 gene mutation associated with autosomal dominant Parkinson’s disease. Lancet 365(9457):412–415 15680456 10.1016/S0140-6736(05)17829-5

[CR51] Di Fonzo A, Dekker M, Montagna P, Baruzzi A, Yonova E, Guedes LC et al (2009a) FBXO7 mutations cause autosomal recessive, early-onset parkinsonian-pyramidal syndrome. Neurology 72(3):240–245 19038853 10.1212/01.wnl.0000338144.10967.2b

[CR52] Di Fonzo A, Fabrizio E, Thomas A, Fincati E, Marconi R, Tinazzi M et al (2009b) GIGYF2 mutations are not a frequent cause of familial Parkinson’s disease. Parkinsonism Relat Disord 15(9):703–705 19482505 10.1016/j.parkreldis.2009.05.001

[CR53] Dickson DW, Braak H, Duda JE, Duyckaerts C, Gasser T, Halliday GM et al (2009) Neuropathological assessment of Parkinson’s disease: refining the diagnostic criteria. Lancet Neurol 8(12):1150–1157 19909913 10.1016/S1474-4422(09)70238-8

[CR54] Dufresne AM, Smith RJ (2005) The adapter protein GRB10 is an endogenous negative regulator of insulin-like growth factor signaling. Endocrinology 146(10):4399–4409 16037382 10.1210/en.2005-0150

[CR55] Edvardson S, Cinnamon Y, Ta-Shma A, Shaag A, Yim YI, Zenvirt S et al (2012) A deleterious mutation in DNAJC6 encoding the neuronal-specific clathrin-uncoating co-chaperone auxilin, is associated with juvenile parkinsonism. PLoS ONE 7(5):e36458 22563501 10.1371/journal.pone.0036458 PMC3341348

[CR56] Evangelou E, Maraganore DM, Ioannidis JP (2007) Meta-analysis in genome-wide association datasets: strategies and application in Parkinson disease. PloS ONE 2(2):e196 17332845 10.1371/journal.pone.0000196 PMC1805816

[CR57] Fan J, Ren H, Jia N, Fei E, Zhou T, Jiang P et al (2008a) DJ-1 decreases Bax expression through repressing p53 transcriptional activity. J Biol Chem 283(7):4022–4030 18042550 10.1074/jbc.M707176200

[CR58] Fan J, Ren H, Fei E, Jia N, Ying Z, Jiang P et al (2008b) Sumoylation is critical for DJ-1 to repress p53 transcriptional activity. FEBS Lett 582(7):1151–1156 18339323 10.1016/j.febslet.2008.03.003

[CR59] Follett J, Norwood SJ, Hamilton NA, Mohan M, Kovtun O, Tay S et al (2014) The Vps35 D620N mutation linked to Parkinson’s disease disrupts the cargo sorting function of retromer. Traffic 15(2):230–244 24152121 10.1111/tra.12136

[CR60] Franco-Iborra S, Vila M, Perier C (2016) The Parkinson disease mitochondrial hypothesis: where are we at? Neuroscientist 22(3):266–277 25761946 10.1177/1073858415574600

[CR61] Frederick RL, Shaw JM (2007) Moving mitochondria: establishing distribution of an essential organelle. Traffic 8(12):1668–1675 17944806 10.1111/j.1600-0854.2007.00644.x PMC3739988

[CR62] Fukushima T, Tan X, Luo Y, Kanda H (2011) Serum vitamins and heavy metals in blood and urine, and the correlations among them in Parkinson’s disease patients in China. Neuroepidemiology 36(4):240–244 21677448 10.1159/000328253

[CR63] Funayama M, Hasegawa K, Ohta E, Kawashima N, Komiyama M, Kowa H et al (2005) An LRRK2 mutation as a cause for the parkinsonism in the original PARK8 family. Ann Neurol 57(6):918–921 15880653 10.1002/ana.20484

[CR64] Funayama M, Ohe K, Amo T, Furuya N, Yamaguchi J, Saiki S et al (2015) CHCHD2 mutations in autosomal dominant late-onset Parkinson’s disease: a genome-wide linkage and sequencing study. Lancet Neurol 14(3):274–282 25662902 10.1016/S1474-4422(14)70266-2

[CR65] Fuse A, Furuya N, Kakuta S, Inose A, Sato M, Koike M et al (2015) VPS29–VPS35 intermediate of retromer is stable and may be involved in the retromer complex assembly process. FEBS Lett 589(13):1430–1436 25937119 10.1016/j.febslet.2015.04.040

[CR66] Gao F, Chen D, Si J, Hu Q, Qin Z, Fang M et al (2015) The mitochondrial protein BNIP3L is the substrate of PARK2 and mediates mitophagy in PINK1/PARK2 pathway. Hum Mol Genet 24(9):2528–2538 25612572 10.1093/hmg/ddv017

[CR67] Gasmi M, Brandon EP, Herzog CD, Wilson A, Bishop KM, Hofer EK et al (2007) AAV2-mediated delivery of human neurturin to the rat nigrostriatal system: long-term efficacy and tolerability of CERE-120 for Parkinson’s disease. Neurobiol Dis 27(1):67–76 17532642 10.1016/j.nbd.2007.04.003

[CR68] Gautier CA, Kitada T, Shen J (2008) Loss of PINK1 causes mitochondrial functional defects and increased sensitivity to oxidative stress. Proc Natl Acad Sci USA 105(32):11364–11369 18687901 10.1073/pnas.0802076105 PMC2516271

[CR69] Gegg ME, Cooper JM, Chau KY, Rojo M, Schapira AH, Taanman JW (2010) Mitofusin 1 and mitofusin 2 are ubiquitinated in a PINK1/parkin-dependent manner upon induction of mitophagy. Hum Mol Genet 19(24):4861–4870 20871098 10.1093/hmg/ddq419 PMC3583518

[CR70] Geisler S, Holmström KM, Skujat D, Fiesel FC, Rothfuss OC, Kahle PJ et al (2010) PINK1/Parkin-mediated mitophagy is dependent on VDAC1 and p62/SQSTM1. Nat Cell Biol 12(2):119–131 20098416 10.1038/ncb2012

[CR71] Gibbs JR, van der Brug MP, Hernandez DG, Traynor BJ, Nalls MA, Lai SL et al (2010) Abundant quantitative trait loci exist for DNA methylation and gene expression in human brain. PLoS Genet 6(5):e1000952 20485568 10.1371/journal.pgen.1000952 PMC2869317

[CR72] Gilks WP, Abou-Sleiman PM, Gandhi S, Jain S, Singleton A, Lees AJ et al (2005) A common LRRK2 mutation in idiopathic Parkinson’s disease. Lancet 365(9457):415–416 15680457 10.1016/S0140-6736(05)17830-1

[CR73] Giovannone B, Lee E, Laviola L, Giorgino F, Cleveland KA, Smith RJ (2003) Two novel proteins that are linked to insulin-like growth factor (IGF-I) receptors by the Grb10 adapter and modulate IGF-I signaling. J Biol Chem 278(34):31564–31573 12771153 10.1074/jbc.M211572200

[CR74] Giovannone B, Tsiaras WG, de la Monte S, Klysik J, Lautier C, Karashchuk G et al (2009) GIGYF2 gene disruption in mice results in neurodegeneration and altered insulin-like growth factor signaling. Hum Mol Genet 18(23):4629–4639 19744960 10.1093/hmg/ddp430 PMC2773276

[CR75] Gitler AD, Chesi A, Geddie ML, Strathearn KE, Hamamichi S, Hill KJ et al (2009) α-Synuclein is part of a diverse and highly conserved interaction network that includes PARK9 and manganese toxicity. Nat Genet 41(3):308–315 19182805 10.1038/ng.300 PMC2683786

[CR76] Goker-Alpan O, Schiffmann R, LaMarca M, Nussbaum R, McInerney-Leo A, Sidransky E (2004) Parkinsonism among Gaucher disease carriers. J Med Genet 41(12):937–940 15591280 10.1136/jmg.2004.024455 PMC1735652

[CR77] Grabowski GA (2008) Phenotype, diagnosis, and treatment of Gaucher’s disease. Lancet 372(9645):1263–1271 19094956 10.1016/S0140-6736(08)61522-6

[CR78] Greene AW, Grenier K, Aguileta MA, Muise S, Farazifard R, Haque ME et al (2012) Mitochondrial processing peptidase regulates PINK1 processing, import and Parkin recruitment. EMBO Rep 13(4):378–385 22354088 10.1038/embor.2012.14 PMC3321149

[CR79] Grünewald A, Arns B, Seibler P, Rakovic A, Münchau A, Ramirez A et al (2012) ATP13A2 mutations impair mitochondrial function in fibroblasts from patients with Kufor-Rakeb syndrome. Neurobiol Aging 33(8):e1841-e1847 10.1016/j.neurobiolaging.2011.12.035 22296644

[CR80] Guilarte TR (2010) Manganese and Parkinson’s disease: a critical review and new findings. Environ Health Perspect 118(8):1071 20403794 10.1289/ehp.0901748 PMC2920085

[CR81] Guo Y, Jankovic J, Zhu S, Le W, Song Z, Xie W et al (2009) GIGYF2 Asn56Ser and Asn457Thr mutations in Parkinson disease patients. Neurosci Lett 454(3):209–211 19429085 10.1016/j.neulet.2009.03.039

[CR82] Gusdon AM, Zhu J, Van Houten B, Chu CT (2012) ATP13A2 regulates mitochondrial bioenergetics through macroautophagy. Neurobiol Dis 45(3):962–972 22198378 10.1016/j.nbd.2011.12.015 PMC3291101

[CR83] Gustavsson EK, Trinh J, Guella I, Vilarino-Guell C, Appel-Cresswell S, Stoessl AJ et al (2015) DNAJC13 genetic variants in parkinsonism. Mov Disord 30:273–278 25393719 10.1002/mds.26064

[CR84] Gwinn–Hardy K, Chen J, Liu HC, Liu T, Boss M, Seltzer W et al (2000) Spinocerebellar ataxia type 2 with parkinsonism in ethnic Chinese. Neurology 55(6):800–805 10993999 10.1212/wnl.55.6.800

[CR85] Halbach MV, Stehning T, Damrath E, Jendrach M, Şen NE, Başak AN et al (2015) Both ubiquitin ligases FBXW8 and PARK2 are sequestrated into insolubility by ATXN2 PolyQ expansions, but only FBXW8 expression is dysregulated. PloS One 10(3):e0121089 25790475 10.1371/journal.pone.0121089 PMC4366354

[CR86] Hansen H, Svensson U, Zhu J, Laviola L, Giorgino F, Wolf G et al (1996) Interaction between the Grb10 SH2 domain and the insulin receptor carboxyl terminus. J Biol Chem 271(15):8882–8886 8621530 10.1074/jbc.271.15.8882

[CR87] Hardy J, Lewis P, Revesz T, Lees A, Paisan-Ruiz C (2009) The genetics of Parkinson’s syndromes: a critical review. Curr Opin Genet Dev 19(3):254–265 19419854 10.1016/j.gde.2009.03.008

[CR88] Healy DG, Abou-Sleiman PM, Wood NW (2004) Genetic causes of Parkinson’s disease: UCHL-1. Cell Tissue Res 318(1):189–194 15221445 10.1007/s00441-004-0917-3

[CR89] Healy DG, Falchi M, O’Sullivan SS, Bonifati V, Durr A, Bressman S et al (2008) Phenotype, genotype, and worldwide genetic penetrance of LRRK2-associated Parkinson’s disease: a case-control study. Lancet Neurol 7(7):583–590 18539534 10.1016/S1474-4422(08)70117-0 PMC2832754

[CR90] Heo JM, Ordureau A, Paulo JA, Rinehart J, Harper JW (2015) The PINK1-PARKIN mitochondrial ubiquitylation pathway drives a program of OPTN/NDP52 recruitment and TBK1 activation to promote mitophagy. Mol Cell 60(1):7–20 26365381 10.1016/j.molcel.2015.08.016 PMC4592482

[CR91] Hernandez DG, Reed X, Singleton AB (2016) Genetics in Parkinson disease: Mendelian versus non-Mendelian inheritance. J Neurochem 139(S1):59–74 27090875 10.1111/jnc.13593 PMC5155439

[CR92] Hicks AA, Pétursson H, Jonsson T, Stefánsson H, Johannsdottir HS, Sainz J et al (2002) A susceptibility gene for late-onset idiopathic Parkinson’s disease. Ann Neurol 52(5):549–555 12402251 10.1002/ana.10324

[CR93] Higashi S, Iseki E, Minegishi M, Togo T, Kabuta T, Wada K (2010) GIGYF2 is present in endosomal compartments in the mammalian brains and enhances IGF-1-induced ERK1/2 activation. J Neurochem 115(2):423–437 20670374 10.1111/j.1471-4159.2010.06930.x

[CR94] Hofseth LJ, Hussain SP, Harris CC (2004) p53: 25 years after its discovery. Trends Pharmacol Sci 25(4):177–181 15116721 10.1016/j.tips.2004.02.009

[CR95] Hozumi I, Hasegawa T, Honda A, Ozawa K, Hayashi Y, Hashimoto K et al (2011) Patterns of levels of biological metals in CSF differ among neurodegenerative diseases. J Neurol Sci 303(1):95–99 21292280 10.1016/j.jns.2011.01.003

[CR96] Hristova VA, Beasley SA, Rylett RJ, Shaw GS (2009) Identification of a novel Zn^2+^-binding domain in the autosomal recessive juvenile Parkinson-related E3 ligase parkin. J Biol Chem 284(22):14978–14986 19339245 10.1074/jbc.M808700200 PMC2685680

[CR97] Ibáñez P, Lesage S, Lohmann E, Thobois S, Michele GD, Borg M et al (2006) Mutational analysis of the PINK1 gene in early-onset parkinsonism in Europe and North Africa. Brain 129(3):686–694 16401616 10.1093/brain/awl005

[CR98] Ibáñez P, Lesage S, Janin S, Lohmann E, Durif F, Destée A et al (2009) α-Synuclein gene rearrangements in dominantly inherited parkinsonism: frequency, phenotype, and mechanisms. Arch Neurol 66(1):102–108 19139307 10.1001/archneurol.2008.555

[CR99] Im JY, Lee KW, Woo JM, Junn E, Mouradian MM (2012) DJ-1 induces thioredoxin 1 expression through the Nrf2 pathway. Hum Mol Genet 21(13):3013–3024 22492997 10.1093/hmg/dds131 PMC3373246

[CR100] Ishikawa A, Tsuji S (1996) Clinical analysis of 17 patients in 12 Japanese families with autosomal-recessive type juvenile parkinsonism. Neurology 47(1):160–166 8710071 10.1212/wnl.47.1.160

[CR101] Ishikawa S, Taira T, Niki T, Takahashi-Niki K, Maita C, Maita H et al (2009) Oxidative status of DJ-1-dependent activation of dopamine synthesis through interaction of tyrosine hydroxylase and 4-dihydroxy-L-phenylalanine (L-DOPA) decarboxylase with DJ-1. J Biol Chem 284(42):28832–28844 19703902 10.1074/jbc.M109.019950 PMC2781429

[CR102] Ishikawa S, Taira T, Takahashi-Niki K, Niki T, Ariga H, Iguchi-Ariga SM (2010) Human DJ-1-specific transcriptional activation of tyrosine hydroxylase gene. J Biol Chem 285(51):39718–39731 20938049 10.1074/jbc.M110.137034 PMC3000953

[CR103] Ishikawa S, Tanaka Y, Takahashi-Niki K, Niki T, Ariga H, Iguchi-Ariga SM (2012) Stimulation of vesicular monoamine transporter 2 activity by DJ-1 in SH-SY5Y cells. Biochem Biophys Res Commun 421(4):813–818 22554508 10.1016/j.bbrc.2012.04.095

[CR104] Itoh K, Nakamura K, Iijima M, Sesaki H (2013) Mitochondrial dynamics in neurodegeneration. Trends Cell Biol 23(2):64–71 23159640 10.1016/j.tcb.2012.10.006 PMC3558617

[CR105] Jankovic J (2008) Parkinson’s disease: clinical features and diagnosis. J Neurol Neurosurg Psychiatr 79(4):368–376 10.1136/jnnp.2007.131045 18344392

[CR106] Jenner P (2003) Oxidative stress in Parkinson’s disease. Ann Neurol 53:S3 12666096 10.1002/ana.10483

[CR107] Jenner P (2004) Preclinical evidence for neuroprotection with monoamine oxidase-B inhibitors in Parkinson’s disease. Neurology 63(7 suppl 2):S13-S22 10.1212/wnl.63.7_suppl_2.s13 15477581

[CR108] Jiménez-Jiménez FJ, Fernández-Calle P, Martínez-Vanaclocha M, Herrero E, Molina JA, Vázquez A et al (1992) Serum levels of zinc and copper in patients with Parkinson’s disease. J Neurol Sci 112(1):30–33 1469436 10.1016/0022-510x(92)90127-7

[CR109] Jin SM, Lazarou M, Wang C, Kane LA, Narendra DP, Youle RJ (2010) Mitochondrial membrane potential regulates PINK1 import and proteolytic destabilization by PARL. J Cell Biol 191(5):933–942 21115803 10.1083/jcb.201008084 PMC2995166

[CR110] Jones JM, Datta P, Srinivasula SM, Ji W, Gupta S, Zhang Z et al (2003) Loss of Omi mitochondrial protease activity causes the neuromuscular disorder of mnd2 mutant mice. Nature 425(6959):721–727 14534547 10.1038/nature02052

[CR111] Junn E, Jang WH, Zhao X, Jeong BS, Mouradian MM (2009) Mitochondrial localization of DJ-1 leads to enhanced neuroprotection. J Neurosci Res 87(1):123–129 18711745 10.1002/jnr.21831 PMC2752655

[CR112] Kabeya Y, Mizushima N, Yamamoto A, Oshitani-Okamoto S, Ohsumi Y, Yoshimori T (2004) LC3, GABARAP and GATE16 localize to autophagosomal membrane depending on form-II formation. J Cell Sci 117(13):2805–2812 15169837 10.1242/jcs.01131

[CR113] Kalinderi K, Bostantjopoulou S, Fidani L (2016) The genetic background of Parkinson’s disease: current progress and future prospects. Acta Neurol Scand 134(5):314–326 26869347 10.1111/ane.12563

[CR114] Kane LA, Lazarou M, Fogel AI, Li Y, Yamano K, Sarraf SA et al (2014) PINK1 phosphorylates ubiquitin to activate Parkin E3 ubiquitin ligase activity. J Cell Biol JCB 201402104 10.1083/jcb.201402104 PMC4003245 24751536

[CR115] Kang S, Louboutin J, Datta P, Landel C, Martinez D, Zervos A et al (2013) Loss of HtrA2/Omi activity in non-neuronal tissues of adult mice causes premature aging. Cell Death Differ 20(2):259–269 22976834 10.1038/cdd.2012.117 PMC3554338

[CR116] Kaplitt MG, Feigin A, Tang C, Fitzsimons HL, Mattis P, Lawlor PA et al (2007) Safety and tolerability of gene therapy with an adeno-associated virus (AAV) borne GAD gene for Parkinson’s disease: an open label, phase I trial. Lancet 369(9579):2097–2105 17586305 10.1016/S0140-6736(07)60982-9

[CR117] Kato I, Maita H, Takahashi-Niki K, Saito Y, Noguchi N, Iguchi-Ariga SM et al (2013) Oxidized DJ-1 inhibits p53 by sequestering p53 from promoters in a DNA-binding affinity-dependent manner. Mol Cell Biol 33(2):340–359 23149933 10.1128/MCB.01350-12 PMC3554126

[CR118] Kauther KM, Höft C, Rissling I, Oertel WH, Möller JC (2011) The PLA2G6 gene in early-onset Parkinson’s disease. Mov Disord 26(13):2415–2417 21812034 10.1002/mds.23851

[CR119] Kazlauskaite A, Kondapalli C, Gourlay R, Campbell DG, Ritorto MS, Hofmann K et al (2014) Parkin is activated by PINK1-dependent phosphorylation of ubiquitin at Ser65. Biochem J 460(1):127–141 24660806 10.1042/BJ20140334 PMC4000136

[CR120] Keller MF, Saad M, Bras J, Bettella F, Nicolaou N, Simón-Sánchez J et al (2012) Using genome-wide complex trait analysis to quantify ‘missing heritability’ in Parkinson’s disease. Hum Mol Genet 21(22):4996–5009 22892372 10.1093/hmg/dds335 PMC3576713

[CR121] Kempster PA, Hurwitz B, Lees AJ (2007) A new look at James Parkinson’s essay on the shaking palsy. Neurology 69(5):482–485 17664408 10.1212/01.wnl.0000266639.50620.d1

[CR122] Kensler TW, Wakabayashi N, Biswal S (2007) Cell survival responses to environmental stresses via the Keap1-Nrf2-ARE pathway. Annu Rev Pharmacol Toxicol 47:89–116 16968214 10.1146/annurev.pharmtox.46.120604.141046

[CR123] Kieper N, Holmström KM, Ciceri D, Fiesel FC, Wolburg H, Ziviani E et al (2010) Modulation of mitochondrial function and morphology by interaction of Omi/HtrA2 with the mitochondrial fusion factor OPA1. Exp Cell Res 316(7):1213–1224 20064504 10.1016/j.yexcr.2010.01.005 PMC3063334

[CR124] Kim SJ, Park YJ, Hwang IY, Youdim MB, Park KS, Oh YJ (2012) Nuclear translocation of DJ-1 during oxidative stress-induced neuronal cell death. Free Radic Biol Med 53(4):936–950 22683601 10.1016/j.freeradbiomed.2012.05.035

[CR125] Kinumi T, Kimata J, Taira T, Ariga H, Niki E (2004) Cysteine-106 of DJ-1 is the most sensitive cysteine residue to hydrogen peroxide-mediated oxidation in vivo in human umbilical vein endothelial cells. Biochem Biophys Res Commun 317(3):722–728 15081400 10.1016/j.bbrc.2004.03.110

[CR126] Klein C, Hedrich K, Wellenbrock C, Kann M, Harris J, Marder K et al (2003) Frequency of parkin mutations in late-onset Parkinson’s disease. Ann Neurol 54(3):415–416 12953277 10.1002/ana.10737

[CR127] Kojovic M, Sheerin UM, Rubio-Agusti I, Saha A, Bras J, Gibbons V et al (2012) Young-onset parkinsonism due to homozygous duplication of α-synuclein in a consanguineous family. Mov Disord 27(14):1829–183023283657 10.1002/mds.25199

[CR128] Kondapalli C, Kazlauskaite A, Zhang N, Woodroof HI, Campbell DG, Gourlay R et al (2012) PINK1 is activated by mitochondrial membrane potential depolarization and stimulates Parkin E3 ligase activity by phosphorylating Serine 65. Open Biol 2(5):120080 22724072 10.1098/rsob.120080 PMC3376738

[CR129] Kong SM, Chan BK, Park JS, Hill KJ, Aitken JB, Cottle L et al (2014) Parkinson’s disease-linked human PARK9/ATP13A2 maintains zinc homeostasis and promotes α-Synuclein externalization via exosomes. Hum Mol Genet 23(11):2816–2833 24603074 10.1093/hmg/ddu099

[CR130] Kono S, Shirakawa K, Ouchi Y, Sakamoto M, Ida H, Sugiura T et al (2007) Dopaminergic neuronal dysfunction associated with parkinsonism in both a Gaucher disease patient and a carrier. J Neurol Sci 252(2):181–184 17182061 10.1016/j.jns.2006.10.019

[CR131] Kononenko NL, Haucke V (2015) Molecular mechanisms of presynaptic membrane retrieval and synaptic vesicle reformation. Neuron 85::484–496 25654254 10.1016/j.neuron.2014.12.016

[CR132] Koroglu C, Baysal L, Cetinkaya M, Karasoy H, Tolun A (2013) DNAJC6 is responsible for juvenile parkinsonism with phenotypic variability. Parkinsonism Relat Disord 19:320–324 23211418 10.1016/j.parkreldis.2012.11.006

[CR133] Koyano F, Okatsu K, Kosako H, Tamura Y, Go E, Kimura M et al (2014) Ubiquitin is phosphorylated by PINK1 to activate parkin. Nature 510(7503):162–166 24784582 10.1038/nature13392

[CR134] Krebs CE, Karkheiran S, Powell JC, Cao M, Makarov V, Darvish H et al (2013) The Sac1 domain of SYNJ1 identified mutated in a family with early-onset progressive parkinsonism with generalized seizures. Hum Mutat 34:1200–1207 23804563 10.1002/humu.22372 PMC3790461

[CR135] Kruglyak L, Daly MJ, Reeve-Daly MP, Lander ES (1996) Parametric and nonparametric linkage analysis: a unified multipoint approach. Am J Hum Genet 58(6):1347 8651312 PMC1915045

[CR136] Kumar KR, Weissbach A, Heldmann M, Kasten M, Tunc S, Sue CM et al (2012) Frequency of the D620N mutation in VPS35 in Parkinson disease. Arch Neurol 69(10):1360–1364 22801713 10.1001/archneurol.2011.3367

[CR137] Lander E, Kruglyak L (1995) Genetic dissection of complex traits: guidelines for interpreting and reporting linkage results. Nat Genet 11(3):241–247 7581446 10.1038/ng1195-241

[CR138] Langlais P, Dong LQ, Ramos FJ, Hu D, Li Y, Quon MJ et al (2004) Negative regulation of insulin-stimulated mitogen-activated protein kinase signaling by Grb10. Mol Endocrinol 18(2):350–358 14615605 10.1210/me.2003-0117

[CR139] Laurent-Matha V, Derocq D, Prébois C, Katunuma N, Liaudet-Coopman E (2006) Processing of human cathepsin D is independent of its catalytic function and auto-activation: involvement of cathepsins L and B. J Biochem 139(3):363–371 16567401 10.1093/jb/mvj037 PMC2376303

[CR140] Lautier C, Goldwurm S, Dürr A, Giovannone B, Tsiaras WG, Pezzoli G et al (2008) Mutations in the GIGYF2 (TNRC15) gene at the PARK11 locus in familial Parkinson disease. Am J Hum Genet 82(4):822–833 18358451 10.1016/j.ajhg.2008.01.015 PMC2427211

[CR141] Lazarou M, Jin SM, Kane LA, Youle RJ (2012) Role of PINK1 binding to the TOM complex and alternate intracellular membranes in recruitment and activation of the E3 ligase Parkin. Dev Cell 22(2):320–333 22280891 10.1016/j.devcel.2011.12.014 PMC3288275

[CR142] Leroy E, Boyer R, Auburger G, Leube B, Ulm G, Mezey E et al (1998) The ubiquitin pathway in Parkinson’s disease. Nature 395(6701):451–452 9774100 10.1038/26652

[CR143] Lesage S, Brice A (2009) Parkinson’s disease: from monogenic forms to genetic susceptibility factors. Hum Mol Genet 18(1):48–59 10.1093/hmg/ddp012 19297401

[CR144] Lesage S, Anheim M, Letournel F, Bousset L, Honoré A, Rozas N et al (2013) G51D α-synuclein mutation causes a novel Parkinsonian–pyramidal syndrome. Ann Neurol 73(4):459–471 23526723 10.1002/ana.23894

[CR145] Lesage S, Drouet V, Majounie E, Deramecourt V, Jacoupy M, Nicolas A et al (2016) Loss of VPS13C function in autosomal-recessive parkinsonism causes mitochondrial dysfunction and increases PINK1/Parkin-dependent mitophagy. Am J Hum Genet 98(3):500–513 26942284 10.1016/j.ajhg.2016.01.014 PMC4800038

[CR146] Lill CM, Roehr JT, McQueen MB, Kavvoura FK, Bagade S, Schjeide BMM et al (2012) Comprehensive research synopsis and systematic meta-analyses in Parkinson’s disease genetics: the PDGene database. PLoS Genet 8(3):e1002548 22438815 10.1371/journal.pgen.1002548 PMC3305333

[CR147] Lim ST, Airavaara M, Harvey BK (2010) Viral vectors for neurotrophic factor delivery: a gene therapy approach for neurodegenerative diseases of the CNS. Pharmacol Res 61(1):14–26 19840853 10.1016/j.phrs.2009.10.002 PMC2880921

[CR148] Lin MK, Farrer MJ (2014) Genetics and genomics of Parkinson’s disease. Genome Med 6(6):48 25061481 10.1186/gm566 PMC4085542

[CR149] Lin L, Ozaki T, Takada Y, Kageyama H, Nakamura Y, Hata A et al (2005) Topors, a p53 and topoisomerase I-binding RING finger protein, is a coactivator of p53 in growth suppression induced by DNA damage. Oncogene 24(21):3385–3396 15735665 10.1038/sj.onc.1208554

[CR150] Lin CH, Chen ML, Chen GS, Tai CH, Wu RM (2011) Novel variant Pro143Ala in HTRA2 contributes to Parkinson’s disease by inducing hyperphosphorylation of HTRA2 protein in mitochondria. Hum Genet 130(6):817–827 21701785 10.1007/s00439-011-1041-6 PMC3214265

[CR151] Liu S, Ninan I, Antonova I, Battaglia F, Trinchese F, Narasanna A et al (2004) α-Synuclein produces a long-lasting increase in neurotransmitter release. EMBO J 23(22):4506–4516 15510220 10.1038/sj.emboj.7600451 PMC526467

[CR152] Liu S, Sawada T, Lee S, Yu W, Silverio G, Alapatt P et al (2012a) Parkinson’s disease-associated kinase PINK1 regulates Miro protein level and axonal transport of mitochondria. PLoS Genet 8(3):e1002537 22396657 10.1371/journal.pgen.1002537 PMC3291531

[CR153] Liu L, Feng D, Chen G, Chen M, Zheng Q, Song P et al (2012b) Mitochondrial outer-membrane protein FUNDC1 mediates hypoxia-induced mitophagy in mammalian cells. Nat Cell Biol 14(2):177–185 22267086 10.1038/ncb2422

[CR154] Liu Y, Clegg H, Leslie P, Di J, Tollini L, He Y et al (2015) CHCHD2 inhibits apoptosis by interacting with Bcl-x L to regulate Bax activation. Cell Death Differ 22(6):1035 25476776 10.1038/cdd.2014.194 PMC4423185

[CR155] Lohmann E, Periquet M, Bonifati V, Wood NW, De Michele G, Bonnet AM et al (2003) How much phenotypic variation can be attributed to parkin genotype? Ann Neurol 54(2):176–185 12891670 10.1002/ana.10613

[CR156] Lohmann E, Thobois S, Lesage S, Broussolle E, du Montcel ST, Ribeiro MJ et al (2009) A multidisciplinary study of patients with early-onset PD with and without parkin mutations. Neurology 72(2):110–116 18987353 10.1212/01.wnl.0000327098.86861.d4 PMC2677494

[CR157] Lotharius J, Brundin P (2002) Impaired dopamine storage resulting from α-synuclein mutations may contribute to the pathogenesis of Parkinson’s disease. Hum Mol Genet 11(20):2395–2407 12351575 10.1093/hmg/11.20.2395

[CR158] Lu CS, Chou YHW, Kuo PC, Chang HC, Weng YH (2004) The parkinsonian phenotype of spinocerebellar ataxia type 2. Arch Neurol 61(1):35–38 14732617 10.1001/archneur.61.1.35

[CR159] Lücking CB, Dürr A, Bonifati V, Vaughan J, De Michele G, Gasser T et al (2000) Association between early-onset Parkinson’s disease and mutations in the parkin gene. N Engl J Med 342(21):1560–1567 10824074 10.1056/NEJM200005253422103

[CR160] Mandel H, Saita S, Edvardson S, Jalas C, Shaag A, Goldsher D et al (2016) Deficiency of HTRA2/Omi is associated with infantile neurodegeneration and 3-methylglutaconic aciduria. J Med Genet. 10.1136/jmedgenet-2016-103922 27208207 10.1136/jmedgenet-2016-103922

[CR161] Manfredsson FP, Burger C, Sullivan LF, Muzyczka N, Lewin AS, Mandel RJ (2007) rAAV-mediated nigral human parkin over-expression partially ameliorates motor deficits via enhanced dopamine neurotransmission in a rat model of Parkinson’s disease. Exp Neurol 207(2):289–301 17678648 10.1016/j.expneurol.2007.06.019

[CR162] Maraganore DM, De Andrade M, Elbaz A, Farrer MJ, Ioannidis JP, Krüger R et al (2006) Collaborative analysis of α-synuclein gene promoter variability and Parkinson disease. JAMA 296(6):661–670 16896109 10.1001/jama.296.6.661

[CR163] Martinat C, Shendelman S, Jonason A, Leete T, Beal MF, Yang L et al (2004) Sensitivity to oxidative stress in DJ-1-deficient dopamine neurons: an ES-derived cell model of primary parkinsonism. PLoS Biol 2(11):e327 15502868 10.1371/journal.pbio.0020327 PMC521171

[CR164] Martins LM, Morrison A, Klupsch K, Fedele V, Moisoi N, Teismann P et al (2004) Neuroprotective role of the Reaper-related serine protease HtrA2/Omi revealed by targeted deletion in mice. Mol Cell Biol 24(22):9848–9862 15509788 10.1128/MCB.24.22.9848-9862.2004 PMC525490

[CR165] Mata IF, Lockhart PJ, Farrer MJ (2004) Parkin genetics: one model for Parkinson’s disease. Hum Mol Genet 13:127–133 10.1093/hmg/ddh089 14976155

[CR166] Matsuda N, Sato S, Shiba K, Okatsu K, Saisho K, Gautier CA et al (2010) PINK1 stabilized by mitochondrial depolarization recruits Parkin to damaged mitochondria and activates latent Parkin for mitophagy. J Cell Biol 189(2):211–221 20404107 10.1083/jcb.200910140 PMC2856912

[CR167] Mazzulli JR, Xu YH, Sun Y, Knight AL, McLean PJ, Caldwell GA et al (2011) Gaucher disease glucocerebrosidase and α-synuclein form a bidirectional pathogenic loop in synucleinopathies. Cell 146(1):37–52 21700325 10.1016/j.cell.2011.06.001 PMC3132082

[CR168] McLelland GL, Soubannier V, Chen CX, McBride HM, Fon EA (2014) Parkin and PINK1 function in a vesicular trafficking pathway regulating mitochondrial quality control. EMBO J 33(4):e201385902 10.1002/embj.201385902 PMC3989637 24446486

[CR169] Meissner C, Lorenz H, Weihofen A, Selkoe DJ, Lemberg MK (2011) The mitochondrial intramembrane protease PARL cleaves human Pink1 to regulate Pink1 trafficking. J Neurochem 117(5):856–867 21426348 10.1111/j.1471-4159.2011.07253.x

[CR170] Meray RK, Lansbury PT (2007) Reversible monoubiquitination regulates the Parkinson disease-associated ubiquitin hydrolase UCH-L1. J Biol Chem 282(14):10567–10575 17259170 10.1074/jbc.M611153200

[CR171] Miura E, Hasegawa T, Konno M, Suzuki M, Sugeno N, Fujikake N et al (2014) VPS35 dysfunction impairs lysosomal degradation of α-synuclein and exacerbates neurotoxicity in a Drosophila model of Parkinson’s disease. Neurobiol Dis 71:1–13 25107340 10.1016/j.nbd.2014.07.014

[CR172] Moghadam AK, Vallian J, Vallian S (2017) Molecular characterization of AIPL1 gene region in the Iranian population: application of novel informative haplotypes and detection of mutational founder effect. Genes Genom 39(4):433–443

[CR173] Moisoi N, Fedele V, Edwards J, Martins LM (2014) Loss of PINK1 enhances neurodegeneration in a mouse model of Parkinson’s disease triggered by mitochondrial stress. Neuropharmacology 77:350–357 24161480 10.1016/j.neuropharm.2013.10.009 PMC3878764

[CR174] Morgan NV, Westaway SK, Morton JE, Gregory A, Gissen P, Sonek S et al (2006) PLA2G6, encoding a phospholipase A2, is mutated in neurodegenerative disorders with high brain iron. Nat Genet 38(7):752–754 16783378 10.1038/ng1826 PMC2117328

[CR175] Mullin S, Schapira A (2013) α-Synuclein and mitochondrial dysfunction in Parkinson’s disease. Mol Neurobiol 47(2):587 23361255 10.1007/s12035-013-8394-x PMC4199090

[CR176] Murphy KE, Cottle L, Gysbers AM, Cooper AA, Halliday GM (2013) ATP13A2 (PARK9) protein levels are reduced in brain tissue of cases with Lewy bodies. Acta Neuropathol Commun 1(1):11 24252509 10.1186/2051-5960-1-11 PMC4046687

[CR177] Mutez E, Leprêtre F, Le Rhun E, Larvor L, Duflot A, Mouroux V et al (2011) SNCA locus duplication carriers: from genetics to Parkinson disease phenotypes. Hum Mutat 32(4) 10.1002/humu.21459 21412942

[CR178] Nagakubo D, Taira T, Kitaura H, Ikeda M, Tamai K, Iguchi-Ariga SM et al (1997) DJ-1, a novel oncogene which transforms mouse NIH3T3 cells in cooperation with ras. Biochem Biophys Res Commun 231(2):509–513 9070310 10.1006/bbrc.1997.6132

[CR179] Nalls MA, Pankratz N, Lill CM, Do CB, Hernandez DG, Saad M et al (2014) Large-scale meta-analysis of genome-wide association data identifies six new risk loci for Parkinson’s disease. Nat Genet 46(9):989 25064009 10.1038/ng.3043 PMC4146673

[CR180] Narendra DP, Jin SM, Tanaka A, Suen DF, Gautier CA, Shen J et al (2010) PINK1 is selectively stabilized on impaired mitochondria to activate Parkin. PLoS Biol 8(1):e1000298 20126261 10.1371/journal.pbio.1000298 PMC2811155

[CR181] Narendra D, Walker JE, Youle R (2012) Mitochondrial quality control mediated by PINK1 and Parkin: links to parkinsonism. Cold Spring Harb Perspect Biol 4(11):a011338 23125018 10.1101/cshperspect.a011338 PMC3536340

[CR182] Nelson DE, Randle SJ, Laman H (2013) Beyond ubiquitination: the atypical functions of Fbxo7 and other F-box proteins. Open Biol 3(10):130131 24107298 10.1098/rsob.130131 PMC3814724

[CR183] Nemani VM, Lu W, Berge V, Nakamura K, Onoa B, Lee MK et al (2010) Increased expression of α-synuclein reduces neurotransmitter release by inhibiting synaptic vesicle reclustering after endocytosis. Neuron 65(1):66–79 20152114 10.1016/j.neuron.2009.12.023 PMC3119527

[CR184] Nichols WC, Pankratz N, Hernandez D, Paisán-Ruíz C, Jain S, Halter CA et al (2005) Genetic screening for a single common LRRK2 mutation in familial Parkinson’s disease. Lancet 365(9457):410–412 15680455 10.1016/S0140-6736(05)17828-3

[CR185] Nishikawa K, Li H, Kawamura R, Osaka H, Wang YL, Hara Y et al (2003) Alterations of structure and hydrolase activity of parkinsonism-associated human ubiquitin carboxyl-terminal hydrolase L1 variants. Biochem biophys Res Commun 304(1):176–183 12705903 10.1016/s0006-291x(03)00555-2

[CR186] Nkiliza A, Mutez E, Simonin C, Leprêtre F, Duflot A, Figeac M et al (2016) RNA-binding disturbances as a continuum from spinocerebellar ataxia type 2 to Parkinson disease. Neurobiol Dis 96:312–322 27663142 10.1016/j.nbd.2016.09.014

[CR187] Nowak DM, Pitarque JA, Molinari A, Bejjani BA, Gajecka M (2012) Linkage analysis as an approach for disease-related loci identification. Comput Methods Sci Technol 18:95–101

[CR188] Nuytemans K, Theuns J, Cruts M, Van Broeckhoven C (2010) Genetic etiology of Parkinson disease associated with mutations in the SNCA, PARK2, PINK1, PARK7, and LRRK2 genes: a mutation update. Hum Mutat 31(7):763–780 20506312 10.1002/humu.21277 PMC3056147

[CR189] O’sullivan SS, Williams DR, Gallagher DA, Massey LA, Silveira-Moriyama L, Lees AJ (2008) Nonmotor symptoms as presenting complaints in Parkinson’s disease: a clinicopathological study. Mov Disord 23(1):101–106 17994582 10.1002/mds.21813

[CR190] Okatsu K, Uno M, Koyano F, Go E, Kimura M, Oka T et al (2013) A dimeric PINK1-containing complex on depolarized mitochondria stimulates Parkin recruitment. J Biol Chem 288(51):36372–36384 24189060 10.1074/jbc.M113.509653 PMC3868751

[CR191] Oláhová M, Thompson K, Hardy SA, Barbosa IA, Besse A, Anagnostou ME et al (2017) Pathogenic variants in HTRA2 cause an early-onset mitochondrial syndrome associated with 3-methylglutaconic aciduria. J Inherit Metab Dis 40(1):121 27696117 10.1007/s10545-016-9977-2 PMC5203855

[CR192] Olgiati S, DE Rosa A, Quadri M, Criscuolo C, Breedveld GJ, Picillo M et al (2014) PARK20 caused by SYNJ1 homozygous Arg258Gln mutation in a new Italian family. Neurogenetics 15:183–188 24816432 10.1007/s10048-014-0406-0

[CR193] Olgiati S, Quadri M, Fang MY, Rood J, Saute JA, Chien HF et al (2016) DNAJC6 mutations associated with early-onset Parkinson’s disease. Ann Neurol 79:244–256 26528954 10.1002/ana.24553

[CR194] Osaka H, Wang YL, Takada K, Takizawa S, Setsuie R, Li H et al (2003) Ubiquitin carboxy-terminal hydrolase L1 binds to and stabilizes monoubiquitin in neuron. Hum Mol Genet 12(16):1945–1958 12913066 10.1093/hmg/ddg211

[CR195] Otsu K, Murakawa T, Yamaguchi O (2015) BCL2L13 is a mammalian homolog of the yeast mitophagy receptor Atg32. Autophagy 11(10):1932–1933 26506896 10.1080/15548627.2015.1084459 PMC4824574

[CR196] Ott J, Wang J, Leal SM (2015) Genetic linkage analysis in the age of whole-genome sequencing. Nat Rev Genet 16(5):275–284 25824869 10.1038/nrg3908 PMC4440411

[CR197] Ottolini D, Calì T, Negro A, Brini M (2013) The Parkinson disease-related protein DJ-1 counteracts mitochondrial impairment induced by the tumour suppressor protein p53 by enhancing endoplasmic reticulum-mitochondria tethering. Hum Mol Genet 22(11):2152–2163 23418303 10.1093/hmg/ddt068

[CR198] Paisan-Ruiz C, Bhatia KP, Li A, Hernandez D, Davis M, Wood NW et al (2009) Characterization of PLA2G6 as a locus for dystonia-parkinsonism. Ann Neurol 65(1):19–23 18570303 10.1002/ana.21415 PMC9016626

[CR199] Paisán-Ruiz C, Guevara R, Federoff M, Hanagasi H, Sina F, Elahi E et al (2010) Early-onset L-dopa-responsive parkinsonism with pyramidal signs due to ATP13A2, PLA2G6, FBXO7 and spatacsin mutations. Mov Disord 25(12):1791–1800 20669327 10.1002/mds.23221 PMC6005705

[CR200] Pals P, Van Everbroeck B, Grubben B, Kristina Viaene M, Dom R, van der Linden C et al (2003) Case–control study of environmental risk factors for Parkinson’s disease in Belgium. Eur J Epidemiol 18(12):1133–1142 14758870 10.1023/b:ejep.0000006639.05690.92

[CR201] Pankratz N, Nichols WC, Uniacke SK, Halter C, Rudolph A, Shults C et al (2002) Genome screen to identify susceptibility genes for Parkinson disease in a sample without parkin mutations. Am J Hum Genet 71(1):124–135 12058349 10.1086/341282 PMC384969

[CR202] Pankratz N, Nichols WC, Uniacke SK, Halter C, Murrell J, Rudolph A et al (2003) Genome-wide linkage analysis and evidence of gene-by-gene interactions in a sample of 362 multiplex Parkinson disease families. Hum Mol Genet 12(20):2599–2608 12925570 10.1093/hmg/ddg270

[CR203] Pao KC, Stanley M, Han C, Lai YC, Murphy P, Balk K et al (2016) Probes of ubiquitin E3 ligases enable systematic dissection of parkin activation. Nat Chem Biol 12(5):324–331 26928937 10.1038/nchembio.2045 PMC4909137

[CR204] Parati E, Fetoni V, Geminiani G, Soliveri P, Giovannini P, Testa D et al (1993) Response to L-DOPA in multiple system atrophy. Clin Neuropharmacol 16(2):139–144 8477409 10.1097/00002826-199304000-00006

[CR205] Park J, Lee SB, Lee S, Kim Y, Song S, Kim S et al (2006) Mitochondrial dysfunction in Drosophila PINK1 mutants is complemented by parkin. Nature 441(7097):1157–1161 16672980 10.1038/nature04788

[CR206] Payami H, Nutt J, Gancher S, Bird T, McNeal MG, Seltzer WK et al (2003) SCA2 may present as levodopa-responsive parkinsonism. Mov Disord 18(4):425–429 12671950 10.1002/mds.10375

[CR207] Periquet M, Latouche M, Lohmann E, Rawal N, De Michele G, Ricard S et al (2003) Parkin mutations are frequent in patients with isolated early-onset parkinsonism. Brain 126(6):1271–1278 12764050 10.1093/brain/awg136

[CR208] Petrucelli L, O’Farrell C, Lockhart PJ, Baptista M, Kehoe K, Vink L et al (2002) Parkin protects against the toxicity associated with mutant α-synuclein: proteasome dysfunction selectively affects catecholaminergic neurons. Neuron 36(6):1007–1019 12495618 10.1016/s0896-6273(02)01125-x

[CR209] Pickrell AM, Youle RJ (2015) The roles of PINK1, parkin, and mitochondrial fidelity in Parkinson’s disease. Neuron 85(2):257–273 25611507 10.1016/j.neuron.2014.12.007 PMC4764997

[CR210] Politis M, Oertel WH, Wu K, Quinn NP, Pogarell O, Brooks DJ et al (2011) Graft-induced dyskinesias in Parkinson’s disease: High striatal serotonin/dopamine transporter ratio. Mov Disord 26(11):1997–2003 21611977 10.1002/mds.23743

[CR211] Polymeropoulos MH, Higgins JJ, Golbe LI, Johnson WG, Ide SE, Di Iorio G et al (1996) Mapping of a gene for Parkinson’s disease to chromosome 4q21-q23. Science 274(5290):1197–1199 8895469 10.1126/science.274.5290.1197

[CR212] Polymeropoulos MH, Lavedan C, Leroy E, Ide SE, Dehejia A, Dutra A et al (1997) Mutation in the α-synuclein gene identified in families with Parkinson’s disease. Science 276(5321):2045–2047 9197268 10.1126/science.276.5321.2045

[CR213] Poole AC, Thomas RE, Andrews LA, McBride HM, Whitworth AJ, Pallanck LJ (2008) The PINK1/Parkin pathway regulates mitochondrial morphology. Proc Natl Acad Sci USA 105(5):1638–1643 18230723 10.1073/pnas.0709336105 PMC2234197

[CR214] Poole AC, Thomas RE, Yu S, Vincow ES, Pallanck L (2010) The mitochondrial fusion-promoting factor mitofusin is a substrate of the PINK1/parkin pathway. PloS ONE 5(4):e10054 20383334 10.1371/journal.pone.0010054 PMC2850930

[CR215] Quadri M, Fang MY, Picillo M, Olgiati S, Breedveld GJ, Graafland J et al (2013) Mutation in the SYNJ1 gene associated with autosomal recessive, early-onset parkinsonism. Hum Mutat 34::1208–1215 23804577 10.1002/humu.22373

[CR216] Ragothaman M, Sarangmath N, Chaudhary S, Khare V, Mittal U, Sharma S et al (2004) Complex phenotypes in an Indian family with homozygous SCA2 mutations. Ann Neurol 55(1):130–133 14705123 10.1002/ana.10815

[CR217] Ramirez A, Heimbach A, Gründemann J, Stiller B, Hampshire D, Cid LP et al (2006) Hereditary parkinsonism with dementia is caused by mutations in ATP13A2, encoding a lysosomal type 5 P-type ATPase. Nat Genet 38(10):1184–1191 16964263 10.1038/ng1884

[CR218] Ramírez-Valle F, Braunstein S, Zavadil J, Formenti SC, Schneider RJ (2008) eIF4GI links nutrient sensing by mTOR to cell proliferation and inhibition of autophagy. J Cell Biol 181(2):293–307 18426977 10.1083/jcb.200710215 PMC2315676

[CR219] Randle J, Laman S H (2017) Structure and function of Fbxo7/PARK15 in Parkinson’s disease. Curr Protein Pept Sci 18(7):715–724 26965690 10.2174/1389203717666160311121433

[CR220] Rathke-Hartlieb S, Schlomann U, Heimann P, Meisler MH, Jockusch H, Bartsch JW (2002) Progressive loss of striatal neurons causes motor dysfunction in MND2 mutant mice and is not prevented by Bcl-2. Exp Neurol 175(1):87–97 12009762 10.1006/exnr.2002.7868

[CR221] Rentschler G, Covolo L, Haddad AA, Lucchini RG, Zoni S, Broberg K (2012) ATP13A2 (PARK9) polymorphisms influence the neurotoxic effects of manganese. Neurotoxicology 33(4):697–702 22285144 10.1016/j.neuro.2012.01.007 PMC3997180

[CR222] Ross JP, Dupre N, Dauvilliers Y, Strong S, Ambalavanan A, Spiegelman D et al (2016) Analysis of DNAJC13 mutations in French-Canadian/French cohort of Parkinson’s disease. Neurobiol Aging 45:e13–e17 10.1016/j.neurobiolaging.2016.04.023 27236598

[CR223] Ryan BJ, Hoek S, Fon EA, Wade-Martins R (2015) Mitochondrial dysfunction and mitophagy in Parkinson’s: from familial to sporadic disease. Trends Biochem Sci 40(4):200–210 25757399 10.1016/j.tibs.2015.02.003

[CR224] Saez-Atienzar S, Bonet-Ponce L, Blesa J, Romero F, Murphy M, Jordan J et al (2014) The LRRK2 inhibitor GSK2578215A induces protective autophagy in SH-SY5Y cells: involvement of Drp-1-mediated mitochondrial fission and mitochondrial-derived ROS signaling. Cell Death Dis 5(8):e1368 25118928 10.1038/cddis.2014.320 PMC4454299

[CR225] Saigoh K, Wang YL, Suh JG, Yamanishi T, Sakai Y, Kiyosawa H et al (1999) Intragenic deletion in the gene encoding ubiquitin carboxy-terminal hydrolase in gad mice. Nat Genet 23(1):47–51 10471497 10.1038/12647

[CR226] Sarraf SA, Raman M, Guarani-Pereira V, Sowa ME, Huttlin EL, Gygi SP et al (2013) Landscape of the PARKIN-dependent ubiquitylome in response to mitochondrial depolarization. Nature 496(7445):372–376 23503661 10.1038/nature12043 PMC3641819

[CR227] Satake W, Nakabayashi Y, Mizuta I, Hirota Y, Ito C, Kubo M et al (2009) Genome-wide association study identifies common variants at four loci as genetic risk factors for Parkinson’s disease. Nat Genet 41(12):1303–1307 19915576 10.1038/ng.485

[CR228] Satterfield TF, Pallanck LJ (2006) Ataxin-2 and its Drosophila homolog, ATX2, physically assemble with polyribosomes. Hum Mol Genet 15(16):2523–2532 16835262 10.1093/hmg/ddl173

[CR229] Schapira AH (2009) Neurobiology and treatment of Parkinson’s disease. Trend Pharmacol Sci 30(1):41–47 10.1016/j.tips.2008.10.005 19042040

[CR230] Schapira AH, Jenner P (2011) Etiology and pathogenesis of Parkinson’s disease. Mov Disord 26(6):1049–1055 21626550 10.1002/mds.23732

[CR231] Schapira AH, Olanow CW, Greenamyre JT, Bezard E (2014) Slowing of neurodegeneration in Parkinson’s disease and Huntington’s disease: future therapeutic perspectives. Lancet 384(9942):545–555 24954676 10.1016/S0140-6736(14)61010-2

[CR232] Schneider RJ, Sonenberg N (2007) Translational control in cancer development and progression. Cold Spring Harbor Monogr Ser 48:401

[CR233] Schrag A, Ben-Shlomo Y, Quinn N (2000) Cross sectional prevalence survey of idiopathic Parkinson’s disease and parkinsonism in London. BMJ 321(7252):21–22 10875828 10.1136/bmj.321.7252.21 PMC27420

[CR234] Schreglmann SR, Houlden H (2016) VPS13C—another hint at mitochondrial dysfunction in familial Parkinson’s disease. Mov Disord 31(9):1340–1340 27213732 10.1002/mds.26682

[CR235] Seaman MN (2007) Identification of a novel conserved sorting motif required for retromer-mediated endosome-to-TGN retrieval. J Cell Sci 120(14):2378–2389 17606993 10.1242/jcs.009654

[CR236] Segura-Aguilar J, Paris I, Muñoz P, Ferrari E, Zecca L, Zucca FA (2014) Protective and toxic roles of dopamine in Parkinson’s disease. J Neurochem 129(6):898–915 24548101 10.1111/jnc.12686

[CR237] Shen Q, Yamano K, Head BP, Kawajiri S, Cheung JT, Wang C et al (2014) Mutations in Fis1 disrupt orderly disposal of defective mitochondria. Mol Biol Cell 25(1):145–159 24196833 10.1091/mbc.E13-09-0525 PMC3873885

[CR238] Shimura H, Hattori N, Kubo SI, Mizuno Y, Asakawa S, Minoshima S et al (2000) Familial Parkinson disease gene product, parkin, is a ubiquitin-protein ligase. Nat Genet 25(3):302–305 10888878 10.1038/77060

[CR239] Shinzawa K, Sumi H, Ikawa M, Matsuoka Y, Okabe M, Sakoda S et al (2008) Neuroaxonal dystrophy caused by group VIA phospholipase A2 deficiency in mice: a model of human neurodegenerative disease. J Neurosci 28(9):2212–2220 18305254 10.1523/JNEUROSCI.4354-07.2008 PMC6671850

[CR240] Shiura H, Miyoshi N, Konishi A, Wakisaka-Saito N, Suzuki R, Muguruma K et al (2005) Meg1/Grb10 overexpression causes postnatal growth retardation and insulin resistance via negative modulation of the IGF1R and IR cascades. Biochem Biophys Res Commun 329(3):909–916 15752742 10.1016/j.bbrc.2005.02.047

[CR241] Shojaee S, Sina F, Banihosseini SS, Kazemi MH, Kalhor R, Shahidi GA et al (2008) Genome-wide linkage analysis of a Parkinsonian-pyramidal syndrome pedigree by 500 K SNP arrays. Am J Hum Genet 82(6):1375–1384 18513678 10.1016/j.ajhg.2008.05.005 PMC2427312

[CR242] Sidransky E (2004) Gaucher disease: complexity in a “simple” disorder. Mol Genet Metab 83(1):6–15 15464415 10.1016/j.ymgme.2004.08.015

[CR243] Silvera D, Arju R, Darvishian F, Levine PH, Zolfaghari L, Goldberg J et al (2009) Essential role for eIF4GI overexpression in the pathogenesis of inflammatory breast cancer. Nat Cell Biol 11(7):903–908 19525934 10.1038/ncb1900

[CR244] Simon-Sanchez J, Schulte C, Bras JM, Sharma M, Gibbs JR, Berg D et al (2009) Genome-wide association study reveals genetic risk underlying Parkinson’s disease. Nat Genet 41(12):1308–1312 19915575 10.1038/ng.487 PMC2787725

[CR245] Simón-Sánchez J, Van Hilten JJ, Van De Warrenburg B, Post B, Berendse HW, Arepalli S et al (2011) Genome-wide association study confirms extant PD risk loci among the Dutch. Eur J Hum Genet 19(6):655–661 21248740 10.1038/ejhg.2010.254 PMC3110043

[CR246] Sina F, Shojaee S, Elahi E, Paisán-Ruiz C (2009) R632W mutation in PLA2G6 segregates with dystonia-parkinsonism in a consanguineous Iranian family. Eur J Neurol 16(1):101–104 19087156 10.1111/j.1468-1331.2008.02356.x

[CR247] Singleton A, Farrer M, Johnson J, Singleton A, Hague S, Kachergus J et al (2003) α-Synuclein locus triplication causes Parkinson’s disease. Science 302(5646):841–841 14593171 10.1126/science.1090278

[CR248] Singleton AB, Farrer MJ, Bonifati V (2013) The genetics of Parkinson’s disease: progress and therapeutic implications. Mov Disord 28(1):14–23 23389780 10.1002/mds.25249 PMC3578399

[CR249] Song Z, Chen H, Fiket M, Alexander C, Chan DC (2007) OPA1 processing controls mitochondrial fusion and is regulated by mRNA splicing, membrane potential, and Yme1L. J Cell Biol 178(5):749–755 17709429 10.1083/jcb.200704110 PMC2064540

[CR250] Stafa K, Tsika E, Moser R, Musso A, Glauser L, Jones A et al (2013) Functional interaction of Parkinson’s disease-associated LRRK2 with members of the dynamin GTPase superfamily. Hum Mol Genet 23(8):2055–2077 24282027 10.1093/hmg/ddt600 PMC3959816

[CR251] Stefanis L (2012) α-Synuclein in Parkinson’s disease. Cold Spring Harb Perspect Med 2(2):a009399 22355802 10.1101/cshperspect.a009399 PMC3281589

[CR252] Stenson PD, Mort M, Ball EV, Evans K, Hayden M, Heywood S et al (2017) The Human Gene Mutation Database: towards a comprehensive repository of inherited mutation data for medical research, genetic diagnosis and next-generation sequencing studies. Hum Genet 1–13 10.1007/s00439-017-1779-6 PMC5429360 28349240

[CR253] Stern MB, Lang A, Poewe W (2012) Toward a redefinition of Parkinson’s disease. Mov Disord 27(1):54–60 22252891 10.1002/mds.24051

[CR254] Strauss KM, Martins LM, Plun-Favreau H, Marx FP, Kautzmann S, Berg D et al (2005) Loss of function mutations in the gene encoding Omi/HtrA2 in Parkinson’s disease. Hum Mol Genet 14(15):2099–2111 15961413 10.1093/hmg/ddi215

[CR255] Su YC, Qi X (2013) Inhibition of excessive mitochondrial fission reduced aberrant autophagy and neuronal damage caused by LRRK2 G2019S mutation. Hum Mol Genet 22(22):4545–4561 23813973 10.1093/hmg/ddt301

[CR256] Sulzer D (2007) Multiple hit hypotheses for dopamine neuron loss in Parkinson’s disease. Trend Neurosci 30(5):244–250 17418429 10.1016/j.tins.2007.03.009

[CR257] Suzuki Y, Imai Y, Nakayama H, Takahashi K, Takio K, Takahashi R (2001) A serine protease, HtrA2, is released from the mitochondria and interacts with XIAP, inducing cell death. Mol cell 8(3):613–621 11583623 10.1016/s1097-2765(01)00341-0

[CR258] Taccioli C, Maselli V, Tegnér J, Gomez-Cabrero D, Altobelli G, Emmett W et al (2011) ParkDB: a Parkinson’s disease gene expression database. Database 2011:bar007. 10.1093/database/bar007 21593080 10.1093/database/bar007 PMC3098727

[CR259] Takahashi K, Yamanaka S (2006) Induction of pluripotent stem cells from mouse embryonic and adult fibroblast cultures by defined factors. Cell 126(4):663–676 16904174 10.1016/j.cell.2006.07.024

[CR260] Tan E, Schapira A (2010) Summary of GIGYF2 studies in Parkinson’s disease: the burden of proof. Eur J Neurol 17(2):175–176 19906271 10.1111/j.1468-1331.2009.02834.x

[CR261] Tan J, Zhang T, Jiang L, Chi J, Hu D, Pan Q et al (2011) Regulation of intracellular manganese homeostasis by Kufor-Rakeb syndrome-associated ATP13A2 protein. J Biol Chem 286(34):29654–29662 21724849 10.1074/jbc.M111.233874 PMC3191006

[CR262] Tanaka K, Suzuki T, Hattori N, Mizuno Y (2004) Ubiquitin, proteasome and parkin. BBA-Mol Cell Res 1695(1):235–247 10.1016/j.bbamcr.2004.09.026 15571819

[CR263] Thomas RE, Andrews LA, Burman JL, Lin WY, Pallanck LJ (2014) PINK1-Parkin pathway activity is regulated by degradation of PINK1 in the mitochondrial matrix. PLoS Genet 10(5):e1004279 24874806 10.1371/journal.pgen.1004279 PMC4038460

[CR264] Tofaris GK (2012) Lysosome-dependent pathways as a unifying theme in Parkinson’s disease. Mov Disord 27(11):1364–1369 22927213 10.1002/mds.25136

[CR265] Trempe JF, Sauvé V, Grenier K, Seirafi M, Tang MY, Ménade M et al (2013) Structure of parkin reveals mechanisms for ubiquitin ligase activation. Science 340(6139):1451–1455 23661642 10.1126/science.1237908

[CR266] Tsika E, Glauser L, Moser R, Fiser A, Daniel G, Sheerin UM et al (2014) Parkinson’s disease-linked mutations in VPS35 induce dopaminergic neurodegeneration. Hum Mol Genet 23(17):4621–4638 24740878 10.1093/hmg/ddu178 PMC4119414

[CR267] Valente EM, Abou-Sleiman PM, Caputo V, Muqit MM, Harvey K, Gispert S et al (2004) Hereditary early-onset Parkinson’s disease caused by mutations in PINK1. Science 304(5674):1158–1160 15087508 10.1126/science.1096284

[CR268] Vargas MR, Johnson JA (2009) The Nrf2–ARE cytoprotective pathway in astrocytes. Expert Rev Mol Med 11 10.1017/S1462399409001094 PMC5563256 19490732

[CR269] Vecchione A, Marchese A, Henry P, Rotin D, Morrione A (2003) The Grb10/Nedd4 complex regulates ligand-induced ubiquitination and stability of the insulin-like growth factor I receptor. Mol Cell Biol 23(9):3363–3372 12697834 10.1128/MCB.23.9.3363-3372.2003 PMC153198

[CR270] Velayati A, Yu WH, Sidransky E (2010) The role of glucocerebrosidase mutations in Parkinson disease and Lewy body disorders. Curr Neurol Neurosci Rep 10(3):190 20425034 10.1007/s11910-010-0102-x PMC3529411

[CR271] Velayos-Baeza A, Vettori A, Copley RR, Dobson-Stone C, Monaco A (2004) Analysis of the human VPS13 gene family. Genomics 84(3):536–549 15498460 10.1016/j.ygeno.2004.04.012

[CR272] Venderova K, Park DS (2012) Programmed cell death in Parkinson’s disease. Cold Spring Harb Perspect Med 2(8):a009365 22908196 10.1101/cshperspect.a009365 PMC3405826

[CR273] Verstraeten A, Theuns J, Van Broeckhoven C (2015) Progress in unraveling the genetic etiology of Parkinson disease in a genomic era. Trends Genet 31(3):140–149 25703649 10.1016/j.tig.2015.01.004

[CR274] Vilarino-Guell C, Rajput A, Milnerwood AJ, Shah B, Szu-Tu C, Trinh J et al (2014) DNAJC13 Mutations In Parkinson Disease. Hum Mol Genet 23:1794–1801 24218364 10.1093/hmg/ddt570 PMC3999380

[CR275] Vilariño-Güell C, Wider C, Ross OA, Dachsel JC, Kachergus JM, Lincoln SJ et al (2011) VPS35 mutations in Parkinson disease. Am J Hum Genet 89(1):162–167 21763482 10.1016/j.ajhg.2011.06.001 PMC3135796

[CR276] Vingill S, Brockelt D, Lancelin C, Tatenhorst L, Dontcheva G, Preisinger C et al (2016) Loss of FBXO7 (PARK15) results in reduced proteasome activity and models a parkinsonism-like phenotype in mice. EMBO J 35(18):2008–2025 27497298 10.15252/embj.201593585 PMC5282834

[CR277] Vives-Bauza C, Zhou C, Huang Y, Cui M, de Vries RL, Kim J et al (2010) PINK1-dependent recruitment of Parkin to mitochondria in mitophagy. Proc Natl Acad Sci USA 107(1):378–383 19966284 10.1073/pnas.0911187107 PMC2806779

[CR278] Walle LV, Lamkanfi M, Vandenabeele P (2008) The mitochondrial serine protease HtrA2/Omi: an overview. Cell Death Differ 15(3):453–460 18174901 10.1038/sj.cdd.4402291

[CR279] Wang G, Mao Z (2014) Chaperone-mediated autophagy: roles in neurodegeneration. Transl Neurodegener 3(1):20 25276349 10.1186/2047-9158-3-20 PMC4177711

[CR280] Wang X, Winter D, Ashrafi G, Schlehe J, Wong YL, Selkoe D et al (2011) PINK1 and Parkin target Miro for phosphorylation and degradation to arrest mitochondrial motility. Cell 147(4):893–906 22078885 10.1016/j.cell.2011.10.018 PMC3261796

[CR281] Wang X, Yan MH, Fujioka H, Liu J, Wilson-Delfosse A, Chen SG et al (2012) LRRK2 regulates mitochondrial dynamics and function through direct interaction with DLP1. Hum Mol Genet 21(9):1931–1944 22228096 10.1093/hmg/dds003 PMC3315202

[CR282] Whone AL, Watts RL, Stoessl AJ, Davis M, Reske S, Nahmias C et al (2003) Slower progression of Parkinson’s disease with ropinirole versus levodopa: the REAL-PET study. Ann Neurol 54(1):93–101 12838524 10.1002/ana.10609

[CR283] Williams DR, Hadeed A, al-Din ASN, Wreikat AL, Lees AJ (2005) Kufor Rakeb disease: autosomal recessive, levodopa-responsive parkinsonism with pyramidal degeneration, supranuclear gaze palsy, and dementia. Mov Disord 20(10):1264–1271 15986421 10.1002/mds.20511

[CR284] Winner B, Jappelli R, Maji SK, Desplats PA, Boyer L, Aigner S et al (2011) In vivo demonstration that α-synuclein oligomers are toxic. Proc Natl Acad Sci USA 108(10):4194–4199 21325059 10.1073/pnas.1100976108 PMC3053976

[CR285] Wszolek ZK, Pfeiffer B, Fulgham J, Parisi JE, Thompson B, Uitti RJ et al (1995) Western Nebraska family (family D) with autosomal dominant parkinsonism. Neurology 45(3):502–505 7898705 10.1212/wnl.45.3.502

[CR286] Xilouri M, Brekk OR, Stefanis L (2016) Autophagy and alpha-synuclein: relevance to Parkinson’s disease and related synucleopathies. Mov Disord 31(2):178–192 26813776 10.1002/mds.26477

[CR287] Xu J, Zhong N, Wang H, Elias JE, Kim CY, Woldman I et al (2005) The Parkinson’s disease-associated DJ-1 protein is a transcriptional co-activator that protects against neuronal apoptosis. Hum Mol Genet 14(9):1231–1241 15790595 10.1093/hmg/ddi134

[CR288] Xu Y, Sun Y, Ran H, Quinn B, Witte D, Grabowski G (2011) Accumulation and distribution of α-synuclein and ubiquitin in the CNS of Gaucher disease mouse models. Mol Genet Metab 102(4):436–447 21257328 10.1016/j.ymgme.2010.12.014 PMC3059359

[CR289] Yamano K, Fogel AI, Wang C, van der Bliek AM, Youle RJ (2014) Mitochondrial Rab GAPs govern autophagosome biogenesis during mitophagy. Elife 3:e01612 24569479 10.7554/eLife.01612 PMC3930140

[CR290] Yang Z, Klionsky DJ (2010) Eaten alive: a history of macroautophagy. Nat Cell Biol 12(9):814–822 20811353 10.1038/ncb0910-814 PMC3616322

[CR291] Yang QH, Church-Hajduk R, Ren J, Newton ML, Du C (2003) Omi/HtrA2 catalytic cleavage of inhibitor of apoptosis (IAP) irreversibly inactivates IAPs and facilitates caspase activity in apoptosis. Genes Dev 17(12):1487–1496 12815069 10.1101/gad.1097903 PMC196079

[CR292] Yoshida S, Hasegawa T, Suzuki M, Sugeno N, Kobayashi J, Ueyama M et al (2018) Parkinson’s DIsease-linked DNAJC13 mutation aggravates alpha-synuclein-induced neurotoxicity through perturbation of endosomal trafficking. Hum Mol Genet 27:823–836 29309590 10.1093/hmg/ddy003

[CR293] Yoshino H, Tomiyama H, Tachibana N, Ogaki K, Li Y, Funayama M et al (2010) Phenotypic spectrum of patients with PLA2G6 mutation and PARK14-linked parkinsonism. Neurology 75(15):1356–1361 20938027 10.1212/WNL.0b013e3181f73649

[CR294] Youle RJ, Narendra DP (2011) Mechanisms of mitophagy. Nat Rev Mol Cell Biol 12(1):9–14 21179058 10.1038/nrm3028 PMC4780047

[CR295] Yu S, Zuo X, Li Y, Zhang C, Zhou M, Zhang YA et al (2004) Inhibition of tyrosine hydroxylase expression in α-synuclein-transfected dopaminergic neuronal cells. Neurosci Lett 367(1):34–39 15308292 10.1016/j.neulet.2004.05.118

[CR296] Yu T, Wang L, Yoon Y (2015) Morphological control of mitochondrial bioenergetics. Front Biosci 20:229 10.2741/4306 PMC4618469 25553448

[CR297] Zabetian CP, Yamamoto M, Lopez AN, Ujike H, Mata IF, Izumi Y et al (2009) LRRK2 mutations and risk variants in Japanese patients with Parkinson’s disease. Mov Disord 24(7):1034–1041 19343804 10.1002/mds.22514 PMC2827255

[CR298] Zavodszky E, Seaman MN, Moreau K, Jimenez-Sanchez M, Breusegem SY, Harbour ME et al (2014) Mutation in VPS35 associated with Parkinson’s disease impairs WASH complex association and inhibits autophagy. Nat Commun 5 10.1038/ncomms4828 PMC4024763 24819384

[CR299] Zhang L, Shimoji M, Thomas B, Moore DJ, Yu SW, Marupudi NI et al (2005) Mitochondrial localization of the Parkinson’s disease related protein DJ-1: implications for pathogenesis. Hum Mol Genet 14(14):2063–2073 15944198 10.1093/hmg/ddi211

[CR300] Zhong N, Kim CY, Rizzu P, Geula C, Porter DR, Pothos EN et al (2006) DJ-1 transcriptionally up-regulates the human tyrosine hydroxylase by inhibiting the sumoylation of pyrimidine tract-binding protein-associated splicing factor. J Biol Chem 281(30):20940–20948 16731528 10.1074/jbc.M601935200

[CR301] Zhou W, Zhu M, Wilson MA, Petsko GA, Fink AL (2006) The oxidation state of DJ-1 regulates its chaperone activity toward α-synuclein. J Mol Biol 356(4):1036–1048 16403519 10.1016/j.jmb.2005.12.030

[CR302] Zhou ZD, Sathiyamoorthy S, Angeles DC, Tan EK (2016) Linking F-box protein 7 and parkin to neuronal degeneration in Parkinson’s disease (PD). Mol Brain 9(1):41 27090516 10.1186/s13041-016-0218-2 PMC4835861

[CR303] Zucca FA, Basso E, Cupaioli FA, Ferrari E, Sulzer D, Casella L, Zecca L (2014) Neuromelanin of the human substantia nigra: an update. Neurotox Res 25(1):13–23 24155156 10.1007/s12640-013-9435-y

